# Multi‐Omics Profiling Reveals Immunomodulatory and Pro‐Regenerative Effects of a Graphene Oxide–Collagen Scaffold in Massive Rotator Cuff Tears

**DOI:** 10.1002/advs.202523821

**Published:** 2026-05-21

**Authors:** Renwen Wan, Yechuan Deng, Zixin Hu, Yanwei He, Xinting Feng, Jun Ma, Yisheng Chen, Zhijie Zhao, Jie Mei, Zhiheng Lin, Wei Luo, Zhengyuan Fang, Zhufeng Hu, Kunlun Feng, Xinrong Li, Dan Wu, Bowen Han, James Hoi po Hui, Xuanyong Liu, Chen Chen, Gang Chen, Shiyi Chen, Nirong Bao, Jiajun Qiu, Zhiwen Luo

**Affiliations:** ^1^ Department of Sports Medicine Huashan Hospital Fudan University Shanghai China; ^2^ Department of Orthopaedics Jiaxing Key Laboratory of Basic Research and Clinical Translation on Orthopedic Biomaterials The Second Affiliated Hospital of Jiaxing University (Sports Hospital of Jiaxing) Jiaxing P. R. China; ^3^ Fudan University – Dr. Kong Joint Research Center for Sports Medicine and Health Footwear Fudan University Institute of Sports Medicine (Jinqiao Laboratory) Shanghai China; ^4^ State Key Laboratory of High Performance Ceramics Shanghai Institute of Ceramics Chinese Academy of Sciences Shanghai China; ^5^ Artificial Intelligence Innovation and Incubation Institute Fudan University Shanghai China; ^6^ Shanghai Academy of Artificial Intelligence for Science Shanghai China; ^7^ Ningde Municipal Hospital Ningde Normal University Ningde Fujian China; ^8^ Fujian Medical University Ningde Fujian China; ^9^ Fujian Key Laboratory of Toxicant and Drug Toxicology Medical College, Ningde Normal University Ningde Fujian China; ^10^ Department of Plastic and Reconstructive Surgery Shanghai Ninth People's Hospital, Shanghai Jiao Tong University School of Medicine Shanghai China; ^11^ The First Clinical Medicine College Nanjing Medical University Nanjing China; ^12^ Department of Gynecology Longhua Hospital Shanghai University of Traditional Chinese Medicine Shanghai China; ^13^ Department of Gastrointestinal Surgery, Renji Hospital, School of Medicine Shanghai Jiao Tong University Shanghai China; ^14^ Department of Integrative Medicine Huashan Hospital Fudan University Shanghai China; ^15^ Department of Orthopedics Zhongda Hospital Southeast University Nanjing China; ^16^ Department of Acupuncture and Moxibustion Yueyang Hospital of Integrated Chinese and Western Medicine Shanghai University of Traditional Chinese Medicine Shanghai China; ^17^ Department of Orthopaedic Surgery Yong Loo Lin School of Medicine National University of Singapore Singapore; ^18^ Department of Arthroscopic Surgery Shanghai Jiao Tong University Affiliated Sixth People's Hospital Shanghai China; ^19^ Department of Orthopedics, Ward 5, 11th Floor, Surgery Building Nanjing Jinling Hospital Affiliated Hospital of Medical School Nanjing University Xuanwu District Nanjing Jiangsu Province China

**Keywords:** graphene oxide, immunomodulation, massive rotator cuff tear, multi‐omics, stem cell differentiation, tendon‐bone interface

## Abstract

Massive rotator cuff tears (MRCT) remain a clinical challenge, characterized by poor tendon‐bone interface (TBI) healing, severe muscle degeneration, and high postoperative retear rates. Tissue engineering scaffolds offer promising alternatives, yet traditional biomaterials often lack sufficient bioactivity to orchestrate comprehensive tissue regeneration. Herein, we developed a novel graphene oxide (GO)‐engineered porcine type I collagen (GO/Col) scaffold and systematically investigated its therapeutic efficacy and underlying molecular mechanisms via multi‐omics analyses. Comprehensive characterization showed that GO incorporation improved scaffold stability, wettability, and biocompatibility. In vitro, the GO/Col scaffold enhanced mesenchymal stem cell adhesion and proliferation, promoted osteogenic and chondrogenic differentiation, and suppressed adipogenesis. In a macrophage model system, GO/Col was associated with a shift toward a more reparative, anti‐inflammatory phenotype. Using a clinically relevant chronic MRCT rat model, we observed that GO/Col scaffolds significantly improved motor function, biomechanical properties, and tendon‐bone regeneration, while inhibiting muscle fibrosis and fatty infiltration. Mechanistically, integrated transcriptomics, proteomics, and mass cytometry analyses revealed GO‐mediated modulation of critical signaling pathways involved in immune regulation, stem cell differentiation, and tissue regeneration. Notably, GO activated pro‐osteogenic/chondrogenic pathways and anti‐inflammatory signatures, while downregulating adipogenic and pro‐inflammatory pathways. Collectively, these findings support the potential of GO/Col scaffolds as a bioactive tissue‐engineering strategy for chronic MRCT repair.

## Graphical abstract

A graphene oxide/collagen scaffold is developed for chronic massive rotator cuff tear repair. The scaffold improves compressive stability, supports reparative mesenchymal differentiation, and modulates the immune microenvironment. In chronic MRCT models, it reduces muscle degeneration, enhances tendon–bone regeneration, and improves functional recovery, while multi‐omics profiling identifies pathways associated with immune regulation and tissue remodeling.

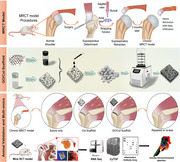

**Abbreviations**
ANOVA: Analysis of Variance
BMD: Bone Mineral Density
BMSC: Bone Marrow Mesenchymal Stem Cell
BrdU: Bromodeoxyuridine
BV/TV: Bone Volume/Total Volume
CCK‐8: Cell Counting Kit‐8
Col: Collagen
Col I: Collagen I
Col II: Collagen II
Col III: Collagen III
cDNA: complementary DNA
CXCL: Chemokine (C‐X‐C motif) Ligand
CyTOF: Cytometry by Time of Flight
DAPI: 4',6‐Diamidino‐2‐Phenylindole
DMEM: Dulbecco's Modified Eagle Medium
dNTP: deoxy‐ribonucleoside Triphosphate
DEPC: Diethyl Pyrocarbonate
EDS: Energy Dispersive Spectrometer
EDTA: Ethylene Diamine Tetraacetic Acid
EDC/NHS: 1‐Ethyl‐3‐(3‐dimethylaminopropyl) carbodiimide/N‐Hydroxysuccinimide
FABP4: Fatty Acid Binding Protein 4
FBS: Foetal Bovine Serum
FTIR: Fourier Transform Infrared Spectroscopy
GAPDH: Glyceraldehyde‐3‐Phosphate Dehydrogenase
GO: Graphene Oxide
GM‐CSF: Granulocyte‐Macrophage Colony‐Stimulating Factor
HE: Hematoxylin‐Eosin
IBMX: 3‐Isobutyl‐1‐methylxanthine
iNOS: inducible Nitric Oxide Synthase
IL: Interleukin
ITS: Insulin‐Transferrin‐Selenium
KEGG: Kyoto Encyclopedia of Genes and Genomes
GSEA: Gene Set Enrichment Analysis
LPS: Lipopolysaccharide
Micro‐CT: Micro‐Computed Tomography
M1: M1 Macrophage
M2: M2 Macrophage
MRCT: Massive Rotator Cuff Tear
mRNA: messenger RNA
NC: Negative Control
NO: Nitric Oxide
NS: No Significance
PBS: Phosphate Buffer Saline
PCA: Principal Component Analysis
PCNA: Proliferating Cell Nuclear Antigen
PPARγ: Peroxisome Proliferator‐Activated Receptor gamma
RCT: Rotator Cuff Tears
RNase A: Ribonuclease A
RNA: Ribose Nucleic Acid
RNA‐Seq: RNA Sequencing
Runx2: Runt‐related Transcription Factor 2
SEM: Scanning Electron Microscope
SPADE: Spanning‐tree Progression Analysis of Density‐normalized Events
Sox‐9: Sex‐determining Region Y Box protein 9
TEM: Transmission Electron Microscope
TBI: Tendon‐bone Interface
TG‐MS: Thermogravimetric Analysis‐Mass Spectrometry
TGF‐β: Transforming Growth Factor‐beta
VEGF: Vascular Endothelial Growth Factor
vi‐SNE: visualization tool for Stochastic Neighbor Embedding
WB: Western Blot
XPS: X‐ray photoelectron spectroscopy
α‐SMA: alpha‐Smooth Muscle Actin
PI: Propidium Iodide

## 1 Introduction

Massive rotator cuff tears (MRCT) represent one of the most challenging conditions encountered in orthopedic surgery and sports medicine, characterized by significant functional impairment, chronic pain, and notably high postoperative retear rates [1, 2]. The rotator cuff, comprising the subscapularis, supraspinatus, infraspinatus, and teres minor muscles, plays a pivotal role in stabilizing the glenohumeral joint and facilitating upper limb motion. Rotator cuff tears (RCT) disrupt this finely balanced dynamic structure, resulting in nocturnal pain, restricted range of motion, and muscle atrophy, thus severely compromising the patients' quality of life [3, 4]. Epidemiological studies indicate that approximately 20% of the general population suffer from varying degrees of RCT, with prevalence rates escalating to nearly 50% among individuals aged 80 years or older [2, 5]. When conservative treatment fails, surgical intervention becomes imperative to restore tendon‐bone interface (TBI) continuity and functionality [6, 7]. However, despite advances in surgical techniques, postoperative retear remains common, with rates varying from 5% to 34%, and alarmingly reaching as high as 90% in cases involving tears larger than 5 cm, classified clinically as MRCT [8, 9]. Consequently, there is an urgent need for innovative strategies aimed at improving TBI regeneration, enhancing mechanical stability, and reducing postoperative complications [10, 11].

Effective tendon‐bone healing involves intricate biological interactions, encompassing a gradient interface comprising tendon, fibrocartilage, mineralized fibrocartilage, and bone tissue [12, 13]. Achieving successful reconstruction of this complex transitional zone poses significant clinical and scientific challenges due to limited regenerative potential and distinct biomechanical requirements [14, 15]. Accumulating evidence underscores the critical roles of bone marrow mesenchymal stem cells (BMSCs) and macrophages in orchestrating the tendon‐bone repair process, primarily through the regulation of cytokine‐mediated signaling cascades [16, 17]. Notably, stem cells facilitate tissue regeneration through differentiation into specific lineages such as osteogenic and chondrogenic cells, whereas macrophages profoundly influence tissue remodeling through polarization into pro‐inflammatory (M1) or anti‐inflammatory (M2) phenotypes [15, 18]. Therefore, strategies that modulate these cellular processes may be critical for improving TBI healing.

Tissue engineering has emerged as a promising approach to address the limitations of conventional surgical methods by providing engineered scaffolds capable of mimicking the native extracellular matrix (ECM) microenvironment [19]. Such scaffolds not only offer mechanical support but also direct cellular behaviors essential for regeneration. Type I collagen (Col I), a major ECM component, is widely utilized in scaffold construction due to its excellent biocompatibility, low immunogenicity, and biomimetic three‐dimensional architecture [20, 21, 22]. Porcine‐derived Col I, closely resembling human collagen structurally and biochemically, further reduces immunogenic risks, positioning itself as an ideal candidate for clinical applications [23, 24, 25]. Nevertheless, pure collagen scaffolds generally lack inherent biological activity to robustly orchestrate cellular differentiation, immune modulation, and sustained regeneration, necessitating functional enhancement through additional bioactive components [26, 27, 28].

Graphene oxide (GO), a two‐dimensional nanomaterial with distinctive physicochemical and biological properties, has recently attracted significant attention in biomedical research, particularly in regenerative medicine [29, 30]. GO possesses an exceptional specific surface area, high mechanical strength, and favorable hydrophilicity, making it an excellent candidate for integration into collagen‐based scaffolds [31, 32]. Moreover, GO has demonstrated intrinsic biological activities, including promoting osteogenic and chondrogenic differentiation, suppressing adipogenesis, and modulating immune responses through the regulation of various cellular signaling pathways [33, 34]. Despite these promising attributes, the comprehensive molecular mechanisms underlying GO‐mediated biological effects and their impact on TBI regeneration remain poorly elucidated, particularly in the context of MRCT repair.

In this study, we developed a graphene oxide‐engineered porcine type I collagen scaffold and evaluated its physicochemical properties, in vitro bioactivity, and in vivo therapeutic efficacy in a chronic MRCT model. We further integrated transcriptomic, proteomic, and cytometric analyses to investigate pathways associated with stem cell regulation, immune remodeling, and tissue regeneration.

By addressing these critical gaps in current knowledge, this study aims to establish the GO/Col scaffold as a promising therapeutic platform with robust bioactivity and clinically relevant regenerative potential, thereby offering novel insights and practical solutions for the challenging management of massive rotator cuff tears.

## 2 Results

**FIGURE 1:**
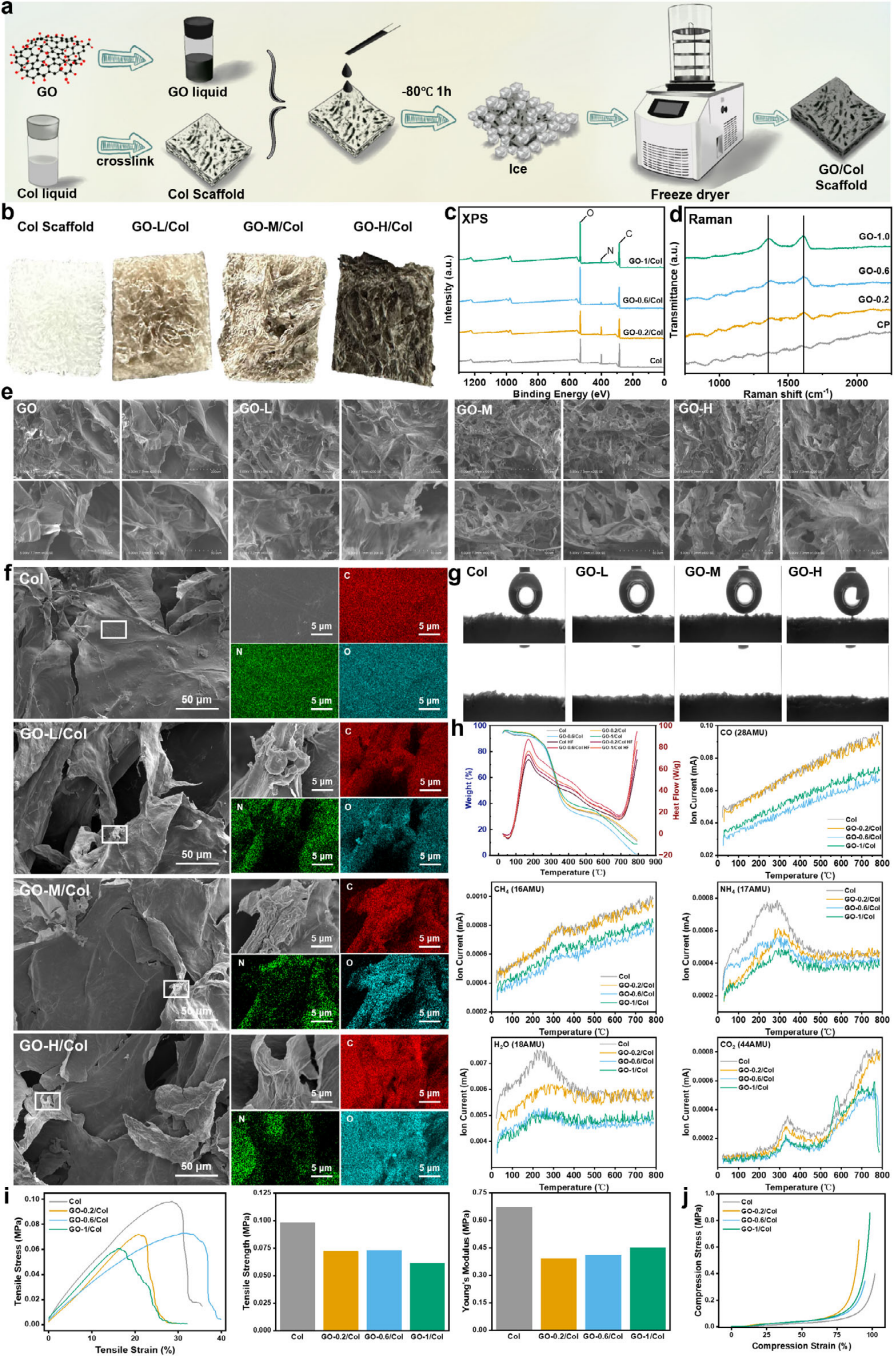
Preparation and characterization of GO/Col scaffolds. (a) Schematic illustration of the synthesis process of the GO/Col scaffold. (b) Macroscopic appearance of scaffolds with different GO concentrations. (c) X‐ray photoelectron spectroscopy (XPS) analysis for surface chemical composition. (d) Raman spectroscopy confirming the characteristic peaks of GO and collagen components. (e) Microstructural morphology (SEM) of scaffolds with different GO concentrations observed under low magnifications; scale bars: 500 µm, 200 µm, 100 µm, and 50 µm. (f) Scanning electron microscopy (SEM) images and elemental mapping of scaffolds with different GO concentrations; scale bars: 50 µm and 5 µm. (g) Sample surfaces before (up) and after (down) water contact angle measurement using sessile drop method. (h) Thermogravimetric analysis (TGA) of GO/Col scaffolds to evaluate their thermal stability. (i) Tensile stress‐strain curves demonstrating the mechanical properties of the GO/Col scaffolds, with bar graphs comparing the ultimate tensile strength and Young's modulus of different scaffold groups. (j) Compressive stress‐strain curves illustrating the compressive behavior of the scaffolds. Col=Col Scaffold; Col‐0.1=Col‐L; Col‐0.6=Col‐M; Col‐1.0=Col‐H.

At the initiation of the experimental protocol, novel graphene oxide/collagen (GO/Col) scaffolds were engineered by physically blending varying concentrations of GO with porcine‐derived Col I sponge matrices using a solution immersion method. Subsequent processing involved either air‐drying or lyophilization (freeze‐drying) (Figure 1a). This fabrication strategy yielded a bioactive collagen‐based scaffold material, which subsequently underwent comprehensive structural and functional characterization to evaluate its potential for MRCT repair. Detailed material characterization was employed to rigorously assess the scaffold's microstructure, chemical composition, and thermal stability (Figure 1 and Figures S1–S3). These foundational analyses established the essential material properties prerequisite for subsequent biological performance evaluation and clinical translation.

### 2.1 Preparation and Characterization of GO/Col Scaffold

The basic synthesis steps for the lyophilized GO/Col scaffold were illustrated in Figure 1a. The scaffold was prepared by mixing monolayer GO solution with Col I, followed by lyophilization. The resulting scaffold displayed a well‐preserved three‐dimensional porous structure with favorable mechanical properties and biocompatibility, suggesting significant potential for biomedical applications. Subsequent evaluations encompassed physicochemical properties, biodegradability, elemental composition, 3D porous structure, and mechanical performance (Figure 1).

GO/Col scaffolds prepared by air‐drying exhibited distinct structural differences compared to untreated scaffolds, as shown in Figure S1a. Scaffolds subjected to air drying revealed wrinkles and cracks, indicative of structural damage during drying. Scanning electron microscopy (SEM) analysis further showed the untreated scaffold's regular porous structure becoming irregular and collapsed with increasing GO concentrations (0.2, 0.6, and 1.0 mg/mL) (Figure 1b). Water contact angle measurements indicated decreased hydrophilicity with higher GO content (Figure S1c). Raman spectroscopy confirmed GO incorporation with intensified characteristic peaks at ∼1350 and 1580 cm^−^^1^ (Figure S1d). X‐ray photoelectron spectroscopy (XPS) demonstrated a carbon‐rich elemental composition with no impurities (Figure S1e). Fourier‐transform infrared spectroscopy (FTIR) maintained typical protein absorption peaks (Figure S1f). Due to structural instability and reduced hydrophilicity, the air‐dried scaffold was deemed unsuitable for further experiments.

Figure 1b presented the macroscopic appearance of freeze‐dried GO/Col scaffolds with increasing GO concentrations, shifting from white to darker shades, correlating visually with GO content. The 3D porous structure remained intact. XPS analysis verified the elemental consistency of carbon (C), nitrogen (N), and oxygen (O), confirming the purity of the composite (Figure 1c). Raman spectroscopy further confirmed GO integration through characteristic peak intensity enhancement at 1350 and 1580 cm^−^^1^ (Figure 1d).

SEM examination (Figure 1e) detailed the microscopic structure of GO/Col scaffolds. Control scaffolds exhibited regular fiber morphology and pronounced porosity. Introducing 0.2 mg/mL GO slightly altered fiber arrangement without compromising porosity. Higher GO concentrations (0.6 and 1.0 mg/mL) caused increased irregularity, minor aggregation, and reduced porosity. Statistically, GO medium and high concentrations significantly reduced porosity compared to controls. SEM/EDS revealed characteristic elements (C, N, O), confirming homogeneous GO distribution and increased roughness and sheet‐like formations at higher GO concentrations (Figure 1f).

All GO/Col scaffolds demonstrated good wettability, exhibiting no significant differences across varying GO concentrations (Figure 1g), implying suitability for biomedical applications requiring high surface hydrophilicity. TG‐MS analysis identified distinct decomposition phases: 150°C–400°C for collagen breakdown and 600°C–800°C for GO decomposition (Figure 1h). GO incorporation notably reduced scaffold moisture content, enhancing thermal stability.

Mechanical testing revealed that the addition of GO did not uniformly improve the tensile properties of the scaffolds (Figure 1i,j). Relative to the Col scaffold, the GO‐containing groups showed reduced ultimate tensile strength, and Young's modulus was likewise lower overall, although it gradually increased from the GO‐L/Col to GO‐H/Col groups. Thus, GO incorporation altered the tensile behavior of the collagen scaffold rather than simply reinforcing it. By contrast, the GO‐containing scaffolds displayed enhanced compressive resistance in the high‐strain region, with the GO‐H/Col group showing the strongest compressive performance.

GO incorporation significantly slowed collagen scaffold degradation in vitro, with lower degradation rates at higher GO concentrations across various time points (1, 7, and 14 days) (Figure S2b,d). GO release rates correlated with scaffold degradation, affirming stable integration and controlled GO dispersion into the medium (Figure S2c). SEM analysis post‐degradation revealed maintained 3D structures, increased surface roughness, and defined microfolds in higher GO groups, further supporting stable composite formation and slowed degradation kinetics (Figure S3).

### 2.2 In Vitro Studies

#### 2.2.1 GO/Col Scaffolds Exhibit Favorable Cytocompatibility

**FIGURE 2:**
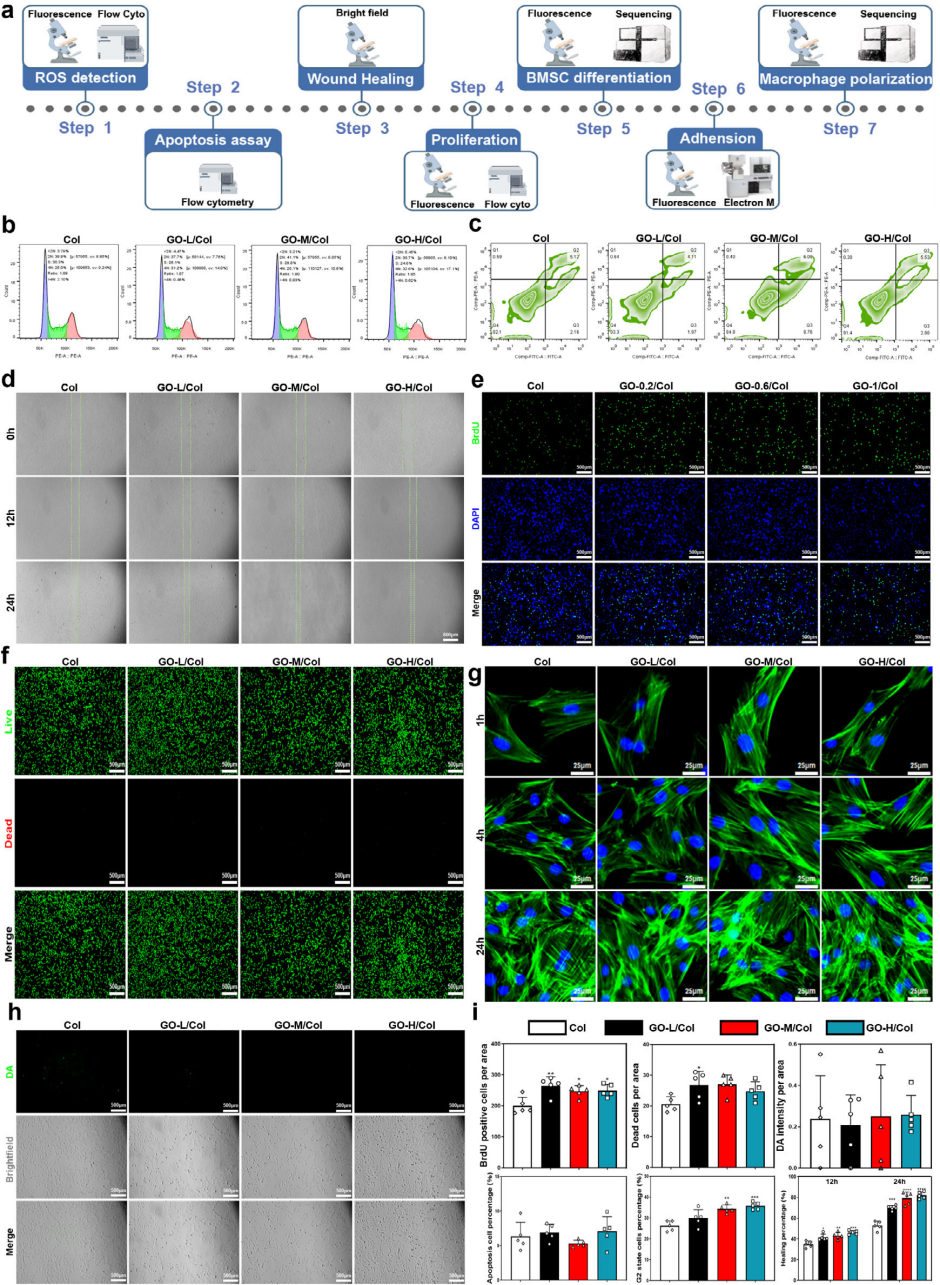
Cytocompatibility of GO/Col scaffolds with BMSCs. (a) Schematic diagram of the in vitro cell experiments. (b–c) Flow cytometry analysis of apoptosis and cell cycle distribution of BMSCs co‐cultured with Col, GO‐L/Col, GO‐M/Col, and GO‐H/Col scaffolds for 1 day. (d) Bright‐field microscopy of BMSC migration; scale bar: 800 µm. (e) BrdU immunofluorescence staining of proliferating BMSCs cultured on different scaffolds at various time points; proliferative cells stained green; scale bar: 500 µm. (f) Live/dead staining of BMSCs cultured on the scaffolds, showing live cells (green) and dead cells (red); scale bar: 500 µm. (g) Fluorescence staining of BMSCs adhered to the scaffold surface at 1, 4, and 24 h; scale bar: 25 µm. (h) Intracellular ROS levels assessed by immunofluorescence staining after 1 day of co‐culture, indicating oxidative stress status; scale bar: 500 µm. (i) Quantitative analysis of proliferation, apoptosis, adhesion, and ROS generation. Data are presented as mean ± SD. For single‐time‐point comparisons among scaffold groups, statistical significance was analyzed by one‐way ANOVA followed by Bonferroni's post hoc test. For time‐course experiments in panels (e) and (g), statistical significance was analyzed by two‐way ANOVA followed by Bonferroni's post hoc test. n = 5 independent samples per group. ns, not significant; *p < 0.05; **p < 0.01; ***p < 0.001; ****p < 0.0001.

Figure 2 comprehensively evaluates the biological interactions between BMSCs and GO/Col scaffolds. Flow cytometric cell cycle analysis showed significantly increased percentages of cells in the G2 phase in the GO‐M and GO‐H groups compared with the control group, suggesting enhanced proliferative activity (Figure 2b,i). In contrast, apoptosis analysis revealed no significant differences in apoptotic rates among groups (Figure 2c,i). Scratch wound‐healing assays further demonstrated that all GO‐containing scaffolds significantly enhanced BMSC migration at both 12 and 24 h relative to the control group, with the GO‐M and GO‐H groups showing greater wound closure than the GO‐L group at 24 h (Figure 2d,i). Consistently, BrdU staining showed significantly increased numbers of BrdU‐positive cells in all GO‐containing groups, indicating enhanced BMSC proliferation (Figure 2e,i). Live/dead staining revealed minimal cell death in all groups, confirming the favorable cytocompatibility of the scaffolds (Figure 2f,i). BMSC adhesion to Col, GO‐L/Col, GO‐M/Col, and GO‐H/Col scaffolds was further examined at 1, 4, and 24 h. At 1 h, cells displayed mainly spherical or polygonal morphologies without obvious differences among groups. By 4 h, limited filopodia and lamellipodia were observed, whereas by 24 h, extensive spreading with abundant protrusions was evident on all scaffold surfaces, indicating that GO incorporation did not impair BMSC adhesion or morphology (Figure 2g,i). ROS staining showed low oxidative stress across all groups (Figure 2h,i). In addition, the CCK‐8 assay confirmed significantly higher cell viability in all GO‐containing groups than in the control group (Figure 2i and Figure S4). Collectively, these results indicate that GO/Col scaffolds enhance BMSC proliferation, migration, and adhesion while maintaining low apoptosis and oxidative stress, demonstrating excellent in vitro biocompatibility.

#### 2.2.2 GO/Col Scaffolds Promote Osteogenic Differentiation of BMSCs

**FIGURE 3:**
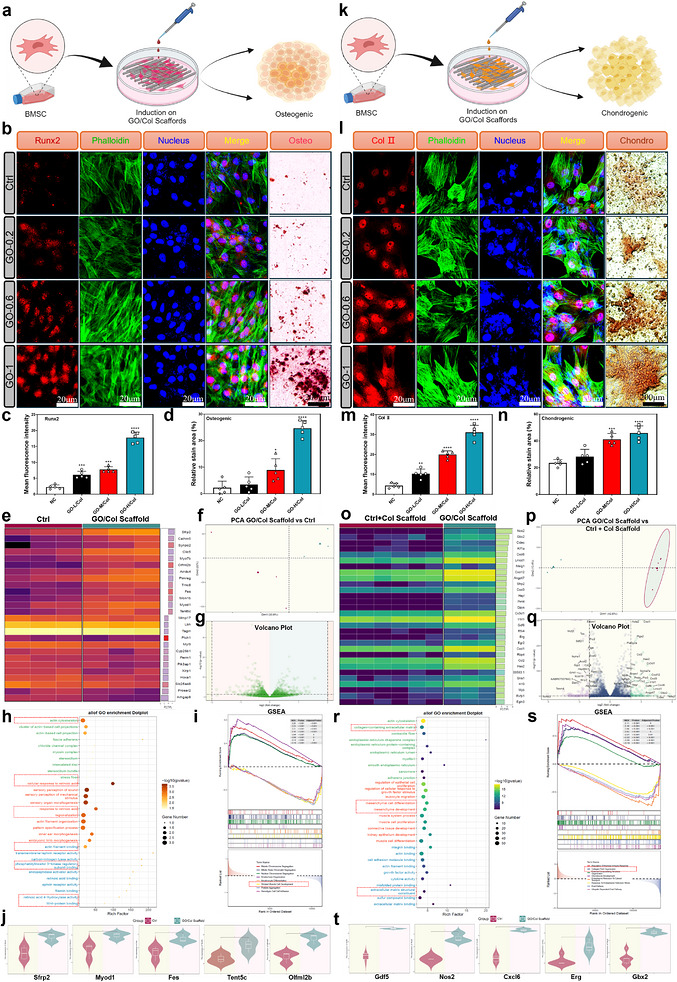
GO/Col scaffolds promote osteogenic and chondrogenic differentiation of BMSCs. (a) Schematic of the osteogenic induction protocol. (b) Immunofluorescence images showing the expression of Runx2 (red) in BMSCs after 48 h of culture on different GO/Col scaffolds; cytoskeleton stained green (phalloidin), nuclei in blue (DAPI). Alizarin Red staining (right) shows calcium deposition after 14 days of osteogenic induction; scale bars: 200 µm and 20 µm. (c–d) Quantification of Runx2 fluorescence intensity and Alizarin Red‐positive area with statistical comparison (n = 5; *p < 0.05; **p < 0.01). (e) Heatmap of differentially expressed genes (DEGs). (f) Principal component analysis (PCA) demonstrating sample clustering. (g) Volcano plot illustrating significant DEGs. (h) Gene ontology enrichment analysis of DEGs. (i) Gene set enrichment analysis (GSEA) highlighting pathways related to osteogenic differentiation. (j) Quantitative PCR (qPCR) results validating the expression of osteogenesis‐related genes (n = 3; *p < 0.05). (k) Schematic of the chondrogenic induction protocol. (l) Immunofluorescence images showing the expression of Col II (red) in BMSCs after 48 h of culture; Safranin O staining (right) indicates proteoglycan deposition after 14 days of chondrogenic induction; scale bars: 200 µm and 20 µm. (m–n) Quantification of Col II fluorescence intensity and Safranin O‐positive area with statistical comparison (n = 5; *p < 0.05; **p < 0.01; ***p < 0.001). (o) Heatmap of DEGs under chondrogenic induction. (p) PCA plot showing the distinct grouping of samples. (q) Volcano plot of chondrogenesis‐related DEGs. (r) GO enrichment analysis of DEGs in chondrogenic induction. (s) GSEA plot highlighting enriched signaling pathways. (t) qPCR results confirming chondrogenic gene expression changes (n = 3; *p < 0.05). Data are presented as mean ± SD. Quantitative data in panels (c), (d), (m), (n), (j), and (t) were analyzed by one‐way ANOVA followed by Bonferroni's post hoc test. n = 5 independent samples per group for panels (c), (d), (m), and (n); n = 3 independent experiments per group for panels (j) and (t). Differentially expressed genes in panels (e–i) and (o–s) were defined using a threshold of |log2(fold change)| > 1 and FDR < 0.05. ns, not significant; *p < 0.05; **p < 0.01; ***p < 0.001; ****p < 0.0001.

The osteogenic differentiation potential of BMSCs cultured on GO/Col scaffolds was systematically evaluated (Figure 3a). Immunofluorescence staining demonstrated a significant enhancement of the osteogenic transcription factor Runx2 with increasing GO concentrations (Figure 3b). Quantitative analysis showed that Runx2 expression was significantly higher in all GO/Col groups compared to the control group (Figure 3c). Additionally, significant differences were observed among GO concentrations, particularly between GO‐H/Col and lower concentration groups. Alizarin Red staining, indicative of calcium deposition and osteogenic differentiation, further validated these findings, revealing markedly larger positively stained areas in GO‐M/Col and GO‐H/Col groups compared to control. The GO‐H/Col group showed significantly higher staining than GO‐L/Col and GO‐M/Col groups (Figure 3b,d).

To explore the underlying mechanism, we performed mRNA sequencing followed by qPCR validation of key genes. The heatmap of differentially expressed genes (DEGs) revealed distinct transcriptional profiles between the control group and the GO/Col group, with notable upregulation of osteogenesis‐associated genes such as Sfrp2, Myod1, Fes, Tent5c, and Olfml2b, all of which are known to play essential roles in skeletal development and cell differentiation (Figure 3e). PCA demonstrated clear separation between the two groups, further supporting the regulatory effect of GO/Col scaffolds on BMSC osteogenesis (Figure 3f). The volcano plot also highlighted a large number of significantly differentially expressed genes between groups (Figure 3g). Gene Ontology enrichment analysis revealed significant enrichment of terms related to osteogenic processes, including “cytoskeleton organization,” “Wnt signaling pathway,” and “response to retinoic acid,” particularly highlighted in red (Figure 3h). These findings support the scaffold's osteoinductive potential at the transcriptional level. GSEA and Kyoto Encyclopedia of Genes and Genomes (KEGG) provided further mechanistic insight. Enriched pathways included those involved in chromosomal segregation and mitotic spindle assembly—processes critical for maintaining genomic stability and supporting cell proliferation and differentiation during osteogenesis. Pathways such as “meiotic chromosome segregation,” “sister chromatid separation,” “nuclear chromosome separation,” and “homotypic cell–cell adhesion” are also essential for bone tissue organization and stability (Figure 3i, Figure S5a,b). Finally, qPCR validation confirmed the RNA‐seq findings, showing significantly elevated expression levels of Sfrp2, Myod1, Fes, Tent5c, and Olfml2b in the GO/Col‐treated group compared to controls (*p* < 0.05) (Figure 3j).

#### 2.2.3 GO/Col Scaffolds Promote Chondrogenic Differentiation of BMSCs

Chondrogenic differentiation was similarly investigated using GO/Col scaffolds (Figure 3k). Immunofluorescence analysis for type II collagen (Col II), a primary marker of chondrogenesis, indicated significantly increased expression correlating positively with GO concentration (Figure 3l,m). Col II fluorescence intensities were markedly elevated in GO‐L/Col, GO‐M/Col, and GO‐H/Col groups compared to controls. Statistically significant differences were also detected between each GO concentration level. Safranin O staining, used to evaluate extracellular cartilage matrix accumulation, demonstrated significantly greater stained areas in GO‐M/Col and GO‐H/Col groups compared to controls. Notably, GO‐M/Col and GO‐H/Col displayed significant differences compared to GO‐L/Col. However, no significant difference was observed between GO‐M/Col and GO‐H/Col groups (Figure 3l,n).

To investigate the mechanism underlying GO/Col‐induced chondrogenic differentiation, we performed mRNA sequencing and qPCR validation. Heatmap, PCA, and volcano plot analyses revealed a distinct transcriptional profile in the GO/Col group, characterized by upregulation of chondrogenesis‐related genes, including Nos2, Gbx2, Gdf6, Cxcl6, and Erg (Figure 3k–q). GO enrichment analysis showed significant enrichment of pathways associated with extracellular matrix collagen, extracellular matrix organization, stem cell differentiation, and stem cell development, while GSEA further highlighted enhanced collagen fibril organization in the GO/Col group (Figure 3r,s and Figure S5c). qPCR validation confirmed the increased expression of Nos2, Gbx2, Gdf6, Cxcl6, and Erg, further supporting the pro‐chondrogenic effect of the GO/Col scaffold (Figure 3t).

#### 2.2.4 GO/Col Scaffold Suppresses Adipogenic Differentiation of BMSCs

**FIGURE 4:**
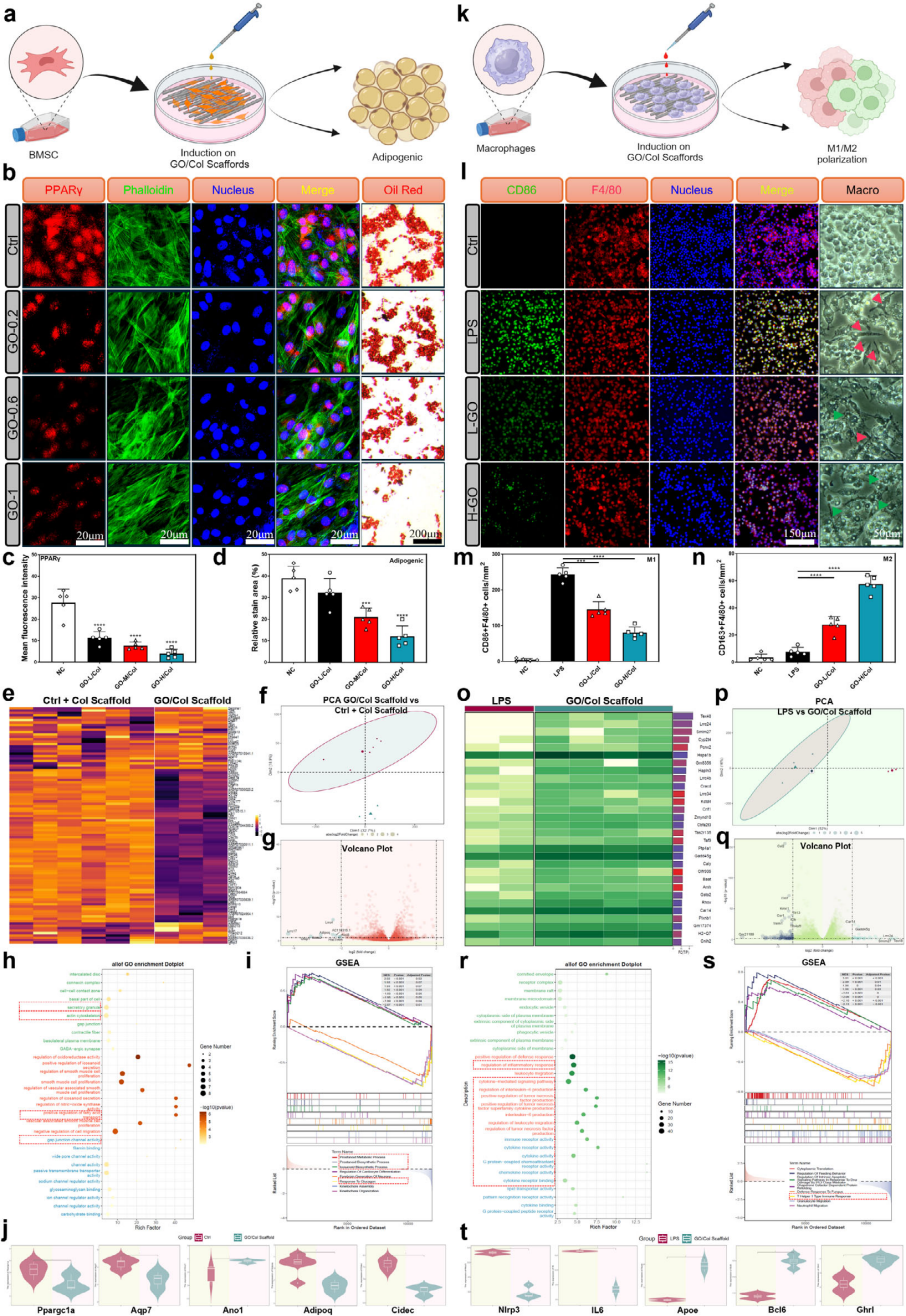
GO/Col scaffolds inhibit adipogenic differentiation of BMSCs and modulate macrophage polarization. (a) Schematic illustration of the adipogenic induction protocol. (b) Immunofluorescence staining showing the expression of PPARγ (red) in BMSCs cultured on different concentrations of GO/Col scaffolds after 48 h; green: cytoskeleton (phalloidin), blue: nucleus (DAPI). Oil Red O staining images (right) illustrate lipid accumulation after 14 days of adipogenic induction; scale bars: 200 µm and 20 µm. (c–d) Quantification of PPARγ fluorescence intensity and Oil Red O‐positive area with intergroup comparison (n = 5; *p < 0.05; **p < 0.01). (e) Heatmap of differentially expressed genes (DEGs) following adipogenic induction. (f) PCA showing sample clustering. (g) Volcano plot of significant DEGs. (h) Gene ontology enrichment analysis of DEGs. (i) GSEA indicating signaling pathways involved in adipogenesis. (j) qPCR validation of adipogenesis‐related genes (n = 3; *p < 0.05). (k) Schematic diagram of macrophage polarization assay. (l) Immunofluorescence staining of RAW264.7 macrophages cultured with GO/Col scaffolds for 24 h, showing the expression of CD86 (green) and F4/80 (red); DAPI stains nuclei (blue). Corresponding bright‐field microscopy images are shown on the right; scale bars: 150 µm and 20 µm. (m,n) Quantification of CD86 and CD163‐positive cells per unit area and intergroup comparison (n = 5; *p < 0.05; **p < 0.01). (o) Heatmap of DEGs in polarized macrophages. (p) PCA plot revealing sample separation. (q) Volcano plot illustrating polarization‐related DEGs. (r) GO enrichment analysis of macrophage DEGs. (s) GSEA showing enriched immune‐related pathways. (t) qPCR results confirming expression changes in polarization‐related genes (n = 3; *p < 0.05). Data are presented as mean ± SD. Quantitative data in panels (c), (d), (m), (n), (j), and (t) were analyzed by one‐way ANOVA followed by Bonferroni's post hoc test. n = 5 independent samples per group for panels (c), (d), (m), and (n); n = 3 independent experiments per group for panels (j) and (t). Differentially expressed genes in panels (e–i) and (o–s) were defined using a threshold of |log2(fold change)| > 1 and FDR < 0.05. ns, not significant; *p < 0.05; **p < 0.01; ***p < 0.001; ****p < 0.0001.

To assess the effect of GO/Col scaffolds on adipogenic differentiation, BMSCs were induced on scaffolds with varying GO concentrations. As shown in Figure 4a, the experimental procedure ensured reproducibility. Staining results revealed a dose‐dependent suppression of adipogenesis. Specifically, expression of the key adipogenic transcription factor PPARγ decreased significantly with increasing GO content, while Oil Red O staining demonstrated reduced lipid accumulation in GO‐treated groups (Figure 4b). Quantitative analysis further confirmed these findings. Fluorescence intensity of PPARγ was significantly lower in all GO/Col groups compared to control. Among GO groups, the high‐concentration scaffold showed the strongest inhibitory effect (Figure 4c,d). Similarly, the area of Oil Red O‐positive staining was significantly reduced in the medium and high GO/Col groups compared to control, indicating diminished lipid deposition (Figure 4b–d). These results demonstrate that GO/Col scaffolds effectively inhibit BMSC adipogenic differentiation in a concentration‐dependent manner.

To further investigate this effect, we performed mRNA sequencing followed by qPCR validation of selected key genes. Differential gene expression analysis revealed a clear distinction between the control and GO/Col groups. Heatmaps showed that genes associated with enhanced adipogenesis—such as Ppargc1a, Aqp7, Adipoq, and Cidec—were significantly downregulated in the GO/Col group, while genes such as Ano1, known to negatively regulate adipogenesis, were upregulated (Figure 4e). PCA confirmed distinct transcriptomic clustering between groups, suggesting that GO/Col scaffold markedly alters gene expression patterns during adipogenic induction (Figure 4f). These findings were further supported by volcano plots, where key adipogenic regulators like Adipoq were significantly suppressed in the GO/Col group (Figure 4g). Gene Ontology enrichment analysis indicated that biological processes such as lipid transport, cytoskeletal regulation, and secretory granule organization were significantly enriched in the GO/Col group (Figure 4h), suggesting these pathways may mediate the scaffold's anti‐adipogenic effects. GSEA and KEGG further highlighted the involvement of prostaglandin metabolism, inositol biosynthesis, and glucagon response pathways (Figure 4i, Figure S6a,b). These metabolic and inflammatory pathways are known to play critical roles in adipocyte differentiation, lipid metabolism, and systemic energy homeostasis, implying that the GO/Col scaffold may inhibit adipogenesis through interference with these signaling cascades. qPCR validation confirmed the transcriptomic findings, demonstrating consistent downregulation of Ppargc1a, Aqp7, Adipoq, and Cidec, and upregulation of Ano1 in GO/Col‐treated cells compared to controls (Figure 4j). These results support the inhibitory effect of the GO/Col scaffold on adipogenic differentiation of BMSCs.

#### 2.2.5 GO/Col Scaffold Modulates Macrophage Polarization Toward the M2 Phenotype

We next investigated the immunomodulatory effects of GO/Col scaffolds on macrophages. As illustrated in the schematic (Figure 4k), RAW264.7 cells were cultured on scaffolds with various GO concentrations. Immunofluorescence staining for CD86 (M1 marker) and CD163 (M2 marker) showed a clear shift toward M2 polarization with increasing GO concentration. GO‐treated macrophages exhibited enhanced CD163 expression and reduced CD86 expression compared to control and LPS groups, suggesting promotion of anti‐inflammatory phenotypes (Figure 4l, Figure S7). Morphological changes consistent with phenotype transition were also observed under microscopy—rounded, pancake‐shaped M2 macrophages increased in the GO/Col groups, while elongated M1 macrophages were reduced (Figure 4l). Quantification of CD86^+^F4/80^+^ cells revealed significantly higher M1 populations in LPS and low‐GO groups compared to control, but these were progressively suppressed in medium and high‐GO groups. In contrast, CD163^+^F4/80^+^ M2 cells were significantly increased in GO‐L and GO‐H groups versus both control and LPS‐treated macrophages, with the high‐GO group showing the greatest effect (Figure 4m,n).

Having demonstrated that the GO/Col scaffold influences macrophage polarization, we next explored the underlying molecular mechanisms using mRNA sequencing, focusing on the scaffold's role in promoting M2 and inhibiting M1 polarization. The heatmap revealed distinct gene expression changes between the GO/Col and control groups. Anti‐inflammatory genes associated with M2 polarization, such as Apoe, Bcl6, and Ghrl, were upregulated, while pro‐inflammatory M1‐related genes, including Nlrp3 and IL6, were downregulated (Figure 4o). PCA analysis showed clear segregation between the two groups, indicating that GO/Col scaffolds induce notable transcriptomic shifts in macrophage phenotype (Figure 4p). Volcano plots identified significantly downregulated pro‐inflammatory genes such as Ccl7 and Ccr1 in the GO/Col group (Figure 4q), reinforcing the scaffold's ability to favor M2 polarization. GO enrichment analysis revealed significant involvement of immune‐related processes, including cytokine activity regulation, inflammatory response, and immune cell chemotaxis (Figure 4r). GSEA and KEGG results showed enrichment of gene sets involved in Th1 immune responses, granulocyte migration, and apoptotic signaling (Figure 4s, Figure S6c), suggesting that GO/Col scaffold exerts immunomodulatory effects through reprogramming of key inflammatory pathways. qPCR results corroborated the sequencing data, showing significantly increased expression of M2 markers (Apoe, Bcl6, Ghrl) and reduced expression of M1 markers (Nlrp3, IL6) in the GO/Col group (Figure 4t). These findings provide further evidence that the GO/Col scaffold promotes M2 macrophage polarization and may contribute to a more regenerative immune microenvironment.

### 2.3 In Vivo Studies

#### 2.3.1 Establishment and Validation of a Chronic MRCT Rat Model and Its Repair Model

**FIGURE 5:**
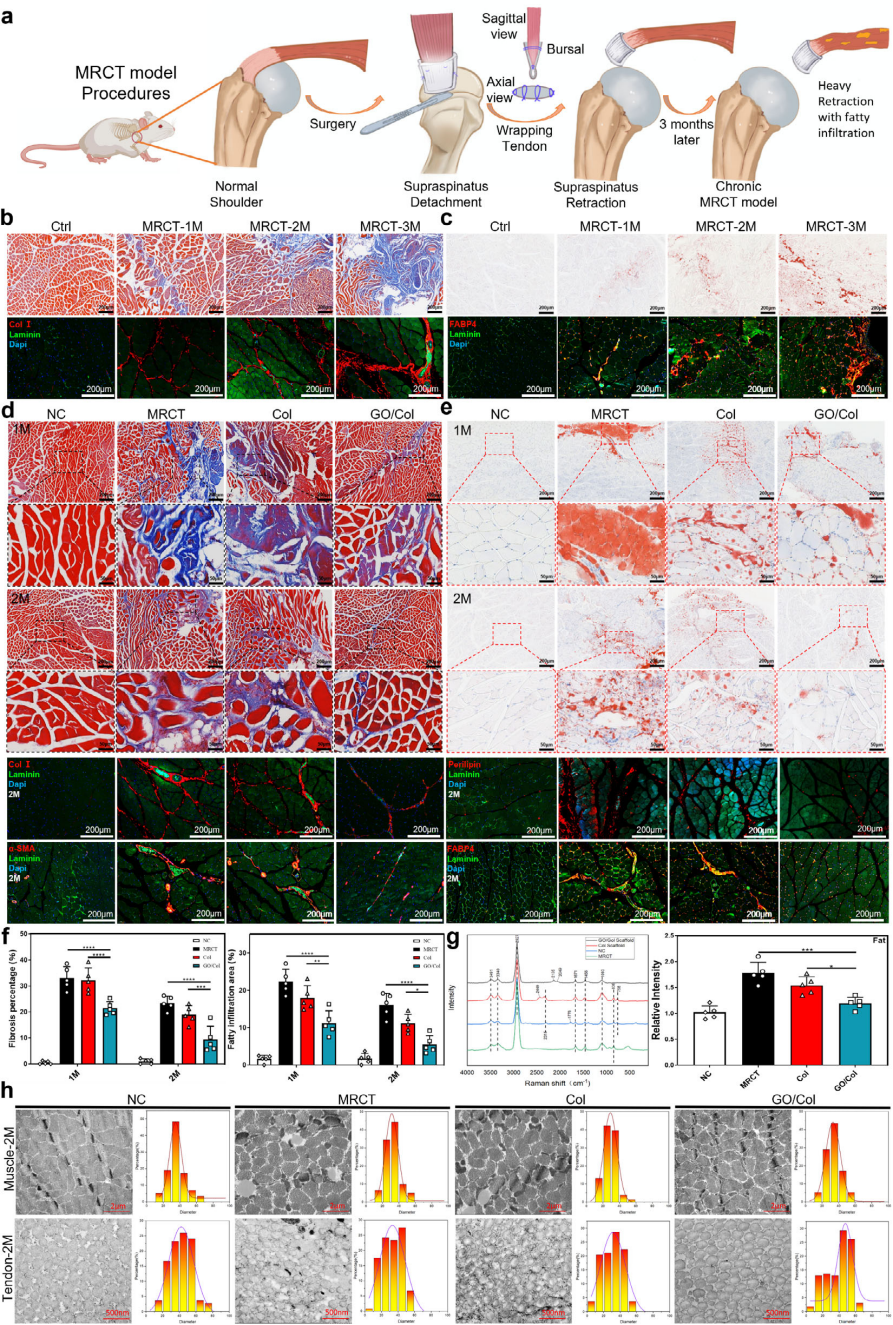
GO/Col scaffolds attenuate supraspinatus muscle degeneration after MRCT repair. (a) Schematic of the chronic massive rotator cuff tear model and surgical repair in rats. (b) Masson's trichrome staining and immunofluorescence staining of collagen I at various time points post‐MRCT to assess muscle fibrosis; scale bar: 200 µm. (c) Oil Red O staining and immunofluorescence for FABP4 showing fat infiltration at different time points post‐MRCT; scale bar: 200 µm. (d) Representative images of supraspinatus muscle fibrosis after MRCT repair: upper panel shows Masson staining at 1 and 2 months; lower panel shows immunofluorescence of fibrotic markers Col I and α‐SMA; scale bars: 200 µm and 50 µm. (e) Representative images of fat infiltration in supraspinatus muscle post‐repair: upper panel shows Oil Red O staining; lower panel shows immunofluorescence of adipogenic markers Perilipin and FABP4; scale bars: 200 µm and 50 µm. (f) Quantitative histological analysis of fibrosis and fat infiltration at 2 months. (g) Assessment of muscle composition and quantification of lipid content post‐MRCT repair. (h) Quantitative analysis of cross‐sectional diameters of muscle and tendon fibers at 2 months; representative transmission electron microscopy images (left) and frequency distribution plots of fiber diameters (right); scale bars: 2 µm and 500 nm. (NC: sham‐operated group; MRCT: repair‐only group; Col: repair + Col scaffold; GO/Col: repair + GO/Col scaffold; n = 5; *p < 0.05; **p < 0.01; ***p < 0.001; ****p < 0.0001). Data are presented as mean ± SD. Quantitative comparisons in panels (f), (g), and (h) were analyzed by one‐way ANOVA followed by Bonferroni's post hoc test. n = 5 animals per group. ns, not significant; *p < 0.05; **p < 0.01; ***p < 0.001; ****p < 0.0001.

Most existing rotator cuff tear repair models are based on acute injury paradigms, which fail to reflect the clinical reality of chronic MRCT. To better simulate clinical scenarios, we established a novel chronic MRCT rat model, as illustrated in Figure 5a. An open surgical approach was adopted to ensure clear visualization and precise manipulation of the supraspinatus tendon (Figure 5a, Figure S8a). 3 months post‐surgery, macroscopic comparisons revealed pronounced differences between the MRCT and sham groups: the supraspinatus muscle in the MRCT group exhibited substantial fatty infiltration, with pale streaks interspersed throughout the muscle, in contrast to the red, healthy appearance observed in controls (Figure S8b). Histological staining further validated the model. Masson's trichrome and Col I immunofluorescence staining demonstrated progressive fibrotic changes in the MRCT group, with markedly increased collagen deposition at 3 months. Oil Red O and FABP4 staining confirmed significant time‐dependent fatty infiltration within the muscle tissue. These findings support the reliability and clinical relevance of this chronic MRCT rat model (Figure 5b,c).

**FIGURE 6:**
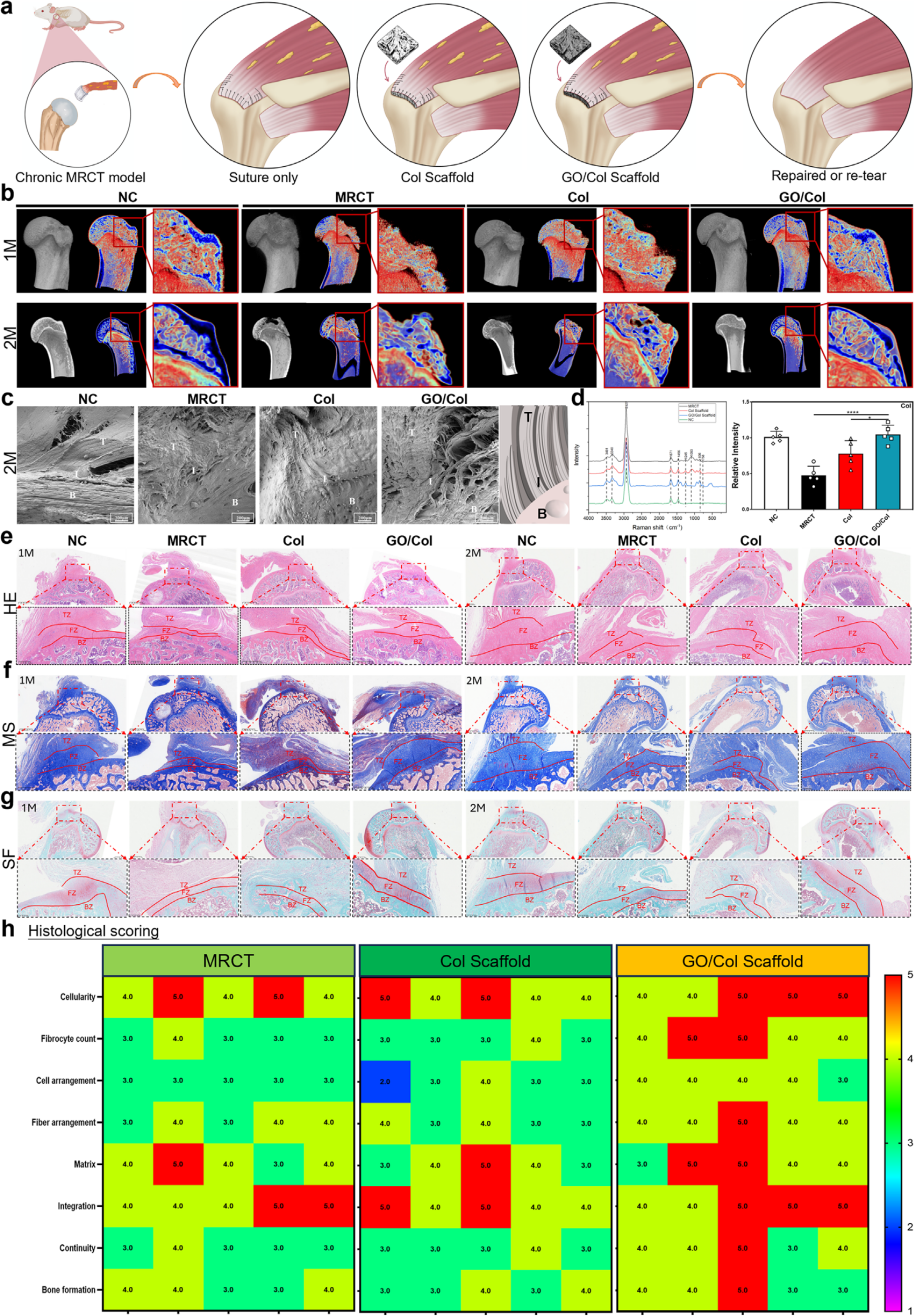
GO/Col scaffolds enhance enthesis remodeling after MRCT repair. (a) Schematic illustration of surgical construction and repair of the chronic MRCT model in rats. (b) Representative Micro‐CT images of the tendon‐to‐bone interface (enthesis) at 4 and 8 weeks post‐repair. (c) Histological analysis of collagen fiber alignment at the enthesis using polarized light microscopy; scale bar: 200 µm. (d) Chemical composition analysis of the tendon‐bone interface at 4 and 8 weeks post‐repair. (e) Hematoxylin and eosin (H&E) staining of the tendon‐bone interface at 4 and 8 weeks post‐repair; scale bars: 200 µm and 50 µm. (f) Masson's trichrome staining of the tendon‐bone interface at 4 and 8 weeks; scale bars: 200 µm and 50 µm. (g) Safranin O/Fast Green staining of the tendon‐bone interface at 4 and 8 weeks; scale bars: 200 µm and 50 µm. (h) Histological scoring of tendon‐bone interface quality.

We further constructed a chronic MRCT repair model to investigate the therapeutic efficacy of biomaterial interventions (Figure 6a). The surgical protocol included debridement of the torn tendon edges and implantation of a GO/Col scaffold, followed by standardized reattachment to the humeral footprint. The complete experimental timeline included macroscopic evaluation, inflammation analysis, functional testing, and imaging at multiple time points (Figure 6a). Representative gross images of the repaired shoulder joints at 1 and 2 months post‐operation confirmed the surgical success and the scaffold placement (Figure S9).

#### 2.3.2 GO/Col Scaffold Attenuates Supraspinatus Muscle Degeneration Post‐MRCT Repair

To investigate the effects of GO/Col scaffolds on muscle degeneration following MRCT repair, histological assessments were conducted at 1 and 2 months postoperatively (Figures 5a and 6a). Masson's trichrome staining revealed significantly reduced fibrosis in the GO/Col group compared to the MRCT and Col‐only scaffold groups at both time points. Immunofluorescence staining further confirmed that the expression of fibrotic markers—Collagen I and α‐SMA—was notably lower in the GO/Col group, indicating effective anti‐fibrotic effects (Figure 5b,c).

Quantitative analysis showed that at 4 weeks post‐surgery, fibrosis levels in the MRCT and Col groups were significantly higher than in the GO/Col group (Figure 5d–f). A similar trend persisted for 8 weeks, although all groups showed a reduction in fibrosis over time. Notably, no significant difference was observed between the MRCT and Col groups at either time point(Figure 5d–f). Oil Red O staining demonstrated reduced fatty infiltration in the GO/Col group, especially at the 2‐month mark. Immunofluorescence analysis of adipogenic markers, including Perilipin and FABP4, confirmed a significant decrease in their expression in the GO/Col group. Quantification further supported that the GO/Col scaffold significantly attenuated fat accumulation compared to the MRCT and Col groups, with no statistical difference from the sham group by week 8(Figure 5d–f).

Raman spectroscopy corroborated these findings, showing a marked reduction in intramuscular lipid content in the GO/Col group relative to the MRCT and Col groups. Importantly, the GO/Col group did not differ statistically from the control group, suggesting a normalization of lipid metabolism (Figure 5g). Transmission electron microscopy revealed that muscle and tendon fiber morphology in the GO/Col group was more organized, with increased fiber diameters closer to those of the sham‐operated group. Quantitative analysis confirmed significantly larger muscle and tendon fiber cross‐sectional diameters in the GO/Col group than in the MRCT and Col groups (Figure 5h), supporting the scaffold's role in preserving tissue structure and promoting regeneration.

#### 2.3.3 GO/Col Scaffold Enhances Enthesis Remodeling Post‐MRCT Repair

Micro‐CT analysis at 1 and 2 months postoperatively demonstrated that bone mineral density (BMD) at the tendon–bone interface was significantly higher in the GO/Col group compared to the MRCT and Col groups at both time points (Figure 6b, Figure S10a). Although all surgical groups exhibited reduced BMD relative to the control, the GO/Col scaffold partially restored bone quality. SEM further revealed improved tendon fiber alignment in the GO/Col group, resembling the organized structure of the sham group. In contrast, the MRCT and Col groups displayed disorganized and dispersed tendon fibers. Fiber orientation angle analysis supported these observations, showing significantly lower angles (i.e., better alignment) in the GO/Col group compared to the MRCT and Col groups (Figure 6c, Figure S10b). Raman spectroscopic evaluation of the enthesis collagen content showed significantly higher collagen levels in the GO/Col group versus MRCT and Col groups, aligning with improved tendon regeneration. The collagen content in the GO/Col group did not significantly differ from the control group, further indicating effective structural restoration (Figure 6d).

At 8 weeks postoperatively, HE staining revealed that the GO/Col scaffold group exhibited reduced inflammatory cell infiltration and neovascularization at the TBI, with more densely and orderly arranged collagen fibers compared to the MRCT and Col groups (Figure 6e). Histological scoring confirmed that although all surgical groups showed inferior tissue organization relative to the sham group, the GO/Col group consistently achieved significantly higher histological scores than the MRCT and Col groups at both 4 and 8 weeks, indicating improved tissue architecture and repair quality (Figure 6h, Figure S10c).

Masson's trichrome staining further demonstrated that the GO/Col scaffold promoted earlier and more organized collagen deposition. At 8 weeks, collagen distribution in the GO/Col group closely resembled that of the normal enthesis (Figure 6f). Safranin O/Fast Green staining showed an increased presence of fibrocartilage in the GO/Col group, again approximating normal tissue structure (Figure 6g). Quantitative analysis revealed significantly higher relative collagen area in the GO/Col group compared to both MRCT and Col groups at 4 and 8 weeks. Notably, by 8 weeks, the GO/Col group reached collagen levels comparable to the sham group, whereas MRCT and Col groups remained significantly deficient (Figure S10d).

#### 2.3.4 GO/Col Scaffold Improves Functional Recovery Post‐MRCT Repair

Restoration of motor function is a central objective in sports medicine. Using advanced behavioral analysis systems, including the CatWalk gait analyzer and grip strength assessment, we evaluated functional outcomes following scaffold implantation. Additionally, biomechanical testing was performed to assess tendon‐to‐bone integration.

**FIGURE 7:**
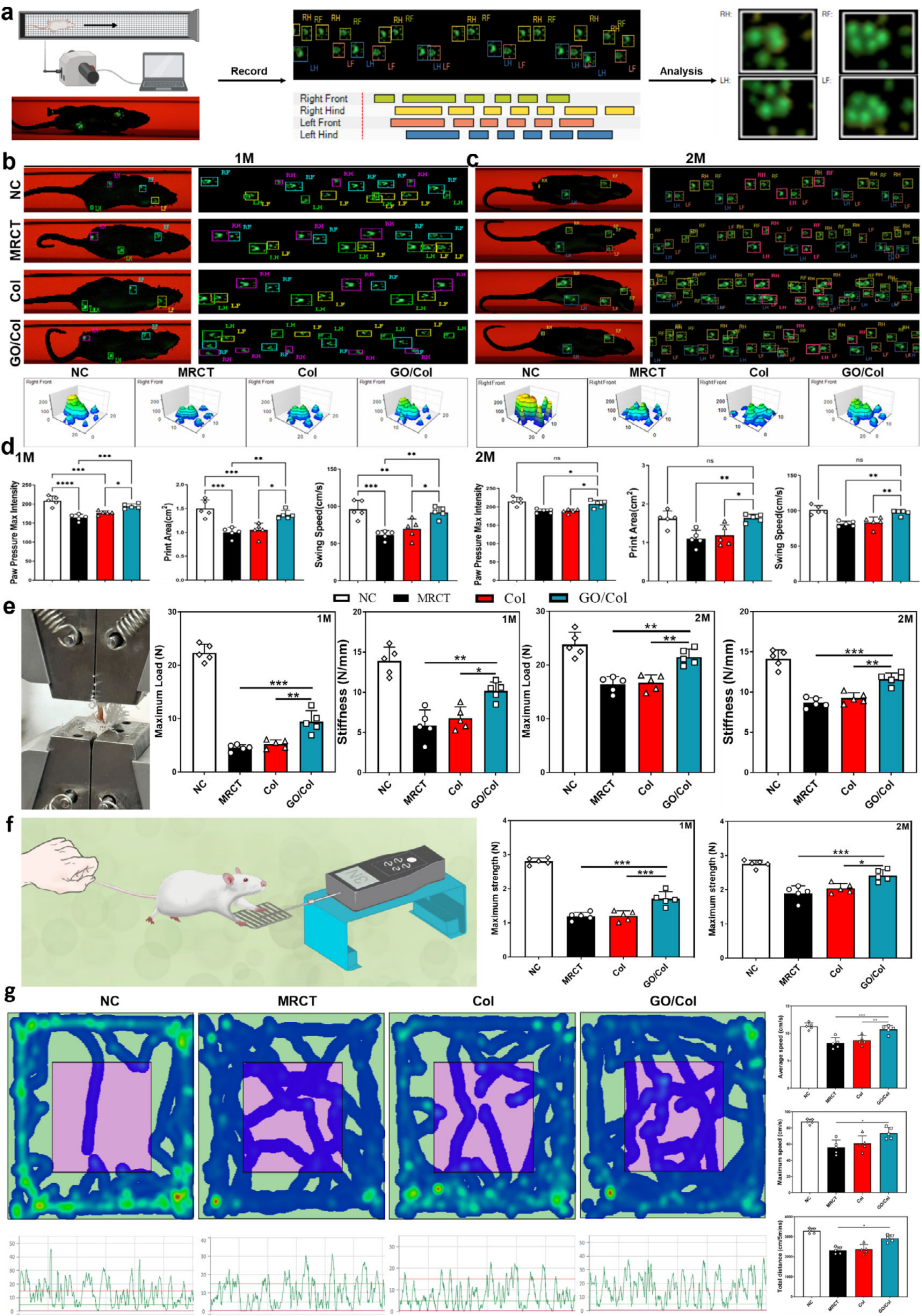
GO/Col scaffold improves functional recovery after MRCT repair. (a) Schematic illustrations of behavioral and gait analysis. (b,c) Representative CatWalk gait footprints of the right hindlimb at different postoperative time points. Dashed lines indicate stance phase (paw contact with ground), and gaps represent swing phase. Corresponding 3D pressure heatmaps of the right forelimb are shown for postoperative weeks 2 and 4. (d) Quantitative analysis of motor function including body speed, footprint length, and right forelimb pressure (n = 5). (NC: sham group; MRCT: repair‐only group; Col: repair + Col scaffold; GO/Col: repair + GO/Col scaffold; ns: no significance; *p < 0.05; **p < 0.01; ***p < 0.001; ****p < 0.0001). (e) Schematic diagram of biomechanical testing of tendon‐to‐bone interface using universal testing machine, and statistical comparisons of biomechanical parameters across groups (n = 5). (f) Schematic of rat forelimb grip strength test and corresponding quantification (n = 5). (g) Open‐field test to assess locomotor capacity. From top to bottom: heatmaps of activity, movement trajectories, and speed variation curves. Right panels: corresponding statistical graphs (n = 5). (NC, MRCT, Col, GO/Col as above; *p < 0.05; **p < 0.01; ***p < 0.001; ****p < 0.0001). Data are presented as mean ± SD. Quantitative behavioral and gait data in panels (d) and (g) were analyzed by two‐way ANOVA followed by Bonferroni's post hoc test, with treatment group and postoperative time as the two factors. Biomechanical and grip strength data in panels (e) and (f) were analyzed by one‐way ANOVA followed by Bonferroni's post hoc test. n = 5 animals per group. ns, not significant; *p < 0.05; **p < 0.01; ***p < 0.001; ****p < 0.0001.

Gait analysis revealed enhanced locomotor coordination and step consistency in the GO/Col group compared to MRCT‐only or Col groups (Figure 7a). Notably, footprint intensity and stride parameters approached those of the sham‐operated group, indicating substantial functional recovery. Three‐dimensional forelimb pressure mapping further confirmed the supportive mechanical role of the GO/Col scaffold. Quantitative analysis showed that at both 4 and 8 weeks post‐surgery, the GO/Col group had significantly higher body speed, paw contact area, and maximum limb pressure than the MRCT and Col groups, with no statistical difference from the control group (Figure 7b–d). These findings indicate that the GO/Col scaffold effectively restores gait parameters and weight‐bearing capacity.

Biomechanical testing using a universal tensile tester demonstrated superior tensile strength at the tendon‐bone interface in the GO/Col group (Figure 7e). At 4 weeks, all experimental groups showed significantly reduced maximum load compared to the sham group, but the GO/Col group achieved a significantly higher maximum load than the MRCT and Col groups. At 8 weeks, the GO/Col group's performance was comparable to the control group and markedly superior to the other two experimental groups, confirming improved tissue integration and mechanical restoration (Figure 7e). Similarly, stiffness measurements mirrored these trends, with the GO/Col group exhibiting significantly enhanced stiffness compared to MRCT and Col groups at both time points, although still lower than controls at 8 weeks. Grip strength testing provided additional evidence of improved muscle function. Rats in the GO/Col group displayed significantly stronger forelimb grip force at 4 and 8 weeks, surpassing MRCT and Col groups and approaching the performance of sham animals (Figure 7f).

Open‐field testing further corroborated the functional improvement. Rats receiving the GO/Col scaffold showed increased locomotor activity, with broader and more frequent movement patterns. Quantitative parameters—including total distance traveled, average velocity, and peak velocity—were significantly improved compared to MRCT and Col groups (Figure 7i). In particular, the GO/Col group exhibited higher peak velocity and total distance than both MRCT and Col groups (*p* < 0.01), confirming enhanced overall physical activity and muscular recovery.

#### 2.3.5 In Vivo Multi‐Omics and Validations Reveal the Mechanisms of GO/Col Scaffold Enhancing TBI Healing

**FIGURE 8:**
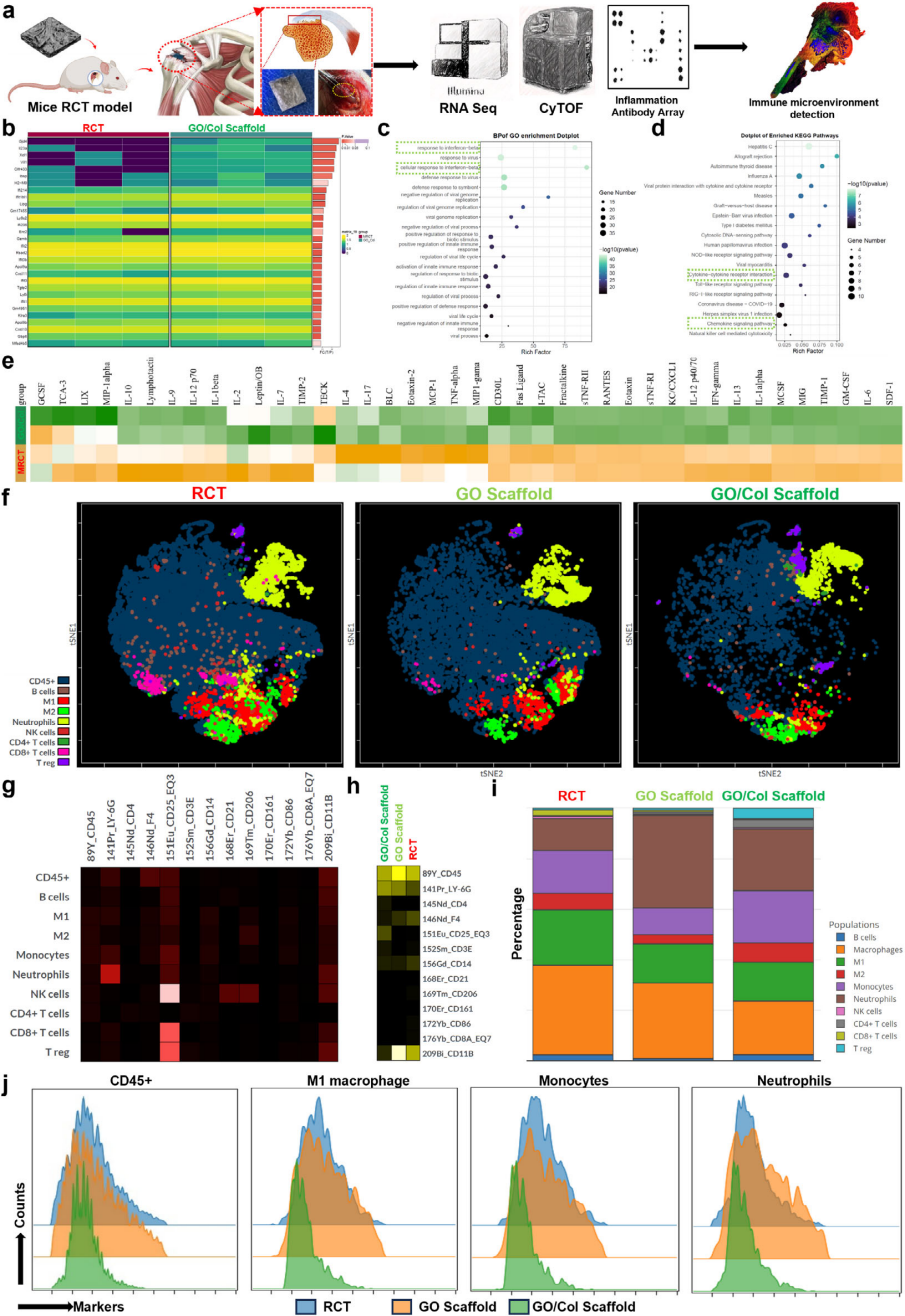
GO/Col scaffold modulates the immune microenvironment to promote TBI regeneration. (a) Schematic of the multi‐omics analysis framework in mice. (b‐d) Heatmap of mRNA sequencing data from TBI and surrounding tissues, with GO and KEGG pathway enrichment analyses. (e) Heatmap of inflammatory cytokine array results from TBI and peri‐lesional tissues. (f–i) CyTOF (cytometry by time‐of‐flight) analysis of TBI and adjacent tissues, including viSNE dimensionality reduction plots, marker expression heatmaps, and cell population proportions. (j) Flow cytometry data for immune cell populations in TBI tissues. Data are presented as mean ± SD where applicable. Differentially expressed genes in panels (b–d) were defined using a threshold of |log2(fold change)| > 1 and FDR < 0.05. Differential inflammatory factors in panel (e) were identified according to the manufacturer's recommended analysis pipeline and significance criteria. Quantitative comparisons of immune cell proportions in panels (f–j) were analyzed by one‐way ANOVA followed by Bonferroni's post hoc test. n = 3 biologically independent pooled samples per group for transcriptomic, CyTOF, and flow cytometry analyses, and n = 2 biologically independent pooled samples per group for the inflammatory cytokine array. ns, not significant; *p < 0.05; **p < 0.01; ***p < 0.001; ****p < 0.0001.

To further explore the biological mechanisms underlying GO/Col scaffold‐induced healing, we applied a multi‐omics approach in a murine RCT model, integrating RNA sequencing, CyTOF, and inflammatory cytokine profiling (Figure 8a).

Transcriptomic analysis revealed distinct gene expression patterns between the RCT and GO/Col groups. Notably, several genes associated with macrophage polarization (e.g., Cxcl10, Xcl1, Gzmb) and stem cell function (e.g., Lipg, Gjd4, Apol9a) were significantly altered in the GO/Col group (Figure 8b). Pathway enrichment analysis (GO, GSEA and KEGG) demonstrated a downregulation of inflammation‐related pathways, including “cytokine–cytokine receptor interaction,” “chemokine signaling,” and “neutrophil chemotaxis,” suggesting that the GO/Col scaffold mitigated the inflammatory response (Figure 8c,d, Figure S11). Protein array analysis confirmed these findings by showing reduced expression of pro‐inflammatory cytokines (e.g., IL‐1α/β, IL‐6, CXCL1, IFN‐γ) in the GO/Col group (Figure 8e).

CyTOF profiling using a 24‐antibody panel and viSNE dimensionality reduction revealed reduced infiltration of M1 macrophages, neutrophils, and T cells in the GO/Col group, with a slight increase in M2 macrophage populations compared to RCT and Col‐only groups (Figure 8f–i, Figure S12). Flow cytometry validation supported these observations, showing decreased numbers of CD45+ leukocytes, M1 macrophages, and neutrophils in the GO/Col group (Figure 8j). Collectively, these data indicate that GO/Col scaffolds modulate the immune microenvironment and suppress excessive post‐injury inflammation.

**FIGURE 9:**
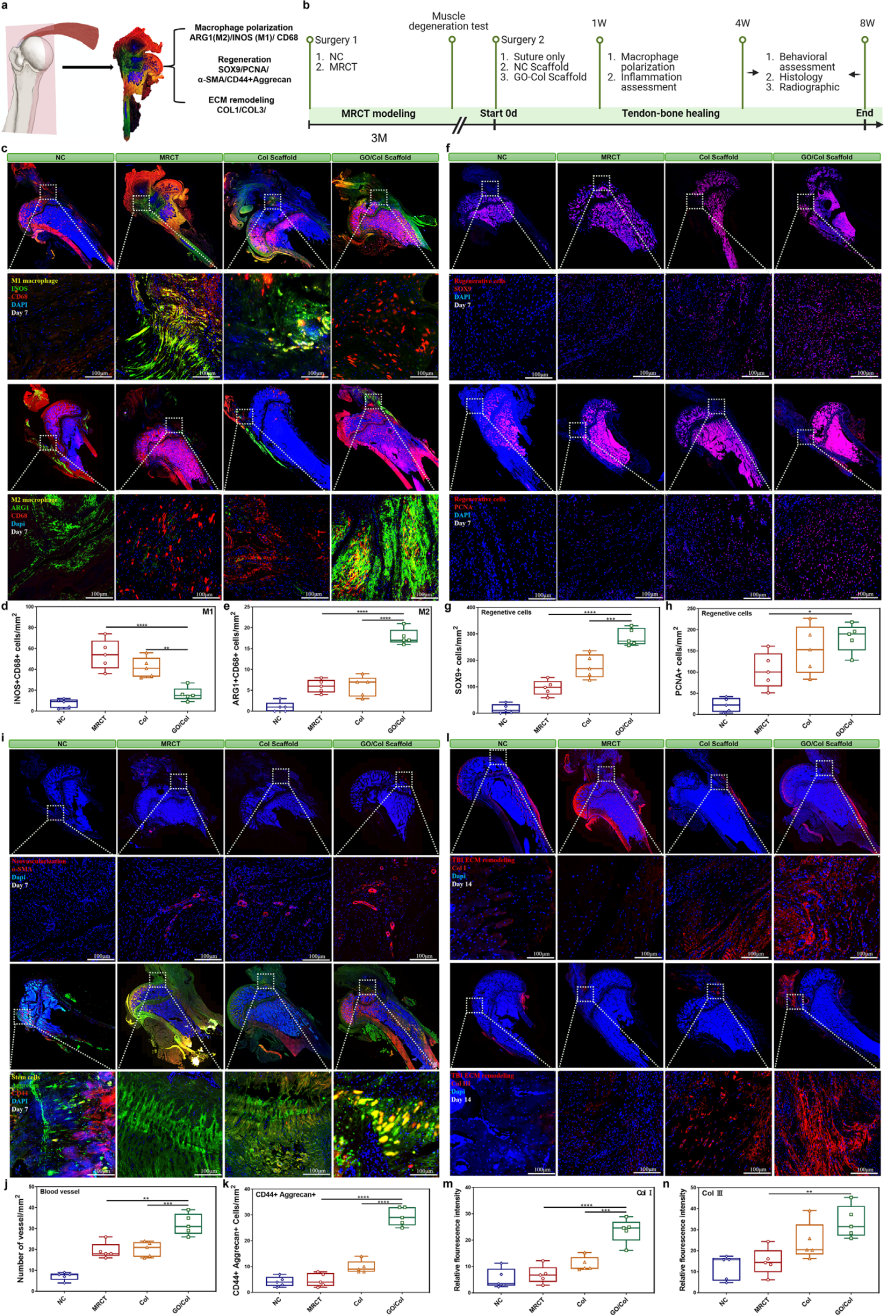
GO/Col scaffold promotes TBI regeneration following MRCT repair. (a,b) Schematic of immunofluorescence staining protocol. (c) Representative immunofluorescence images of M1 and M2 macrophages. M1 macrophages were identified using iNOS (green) and CD68 (red); M2 macrophages were labeled with ARG1 (green) and CD68 (red). DAPI (blue) stains nuclei. Scale bar: 100 µm. (d,e) Quantification of M1 and M2 macrophages per unit area in the TBI region (n = 5; **p < 0.01; ****p < 0.0001). (f) Immunofluorescence images showing SOX9 and PCNA (red) expression, markers of cell regeneration and proliferation, respectively. Nuclei are stained with DAPI (blue); scale bar: 100 µm. (g,h) Quantification of SOX9+ and PCNA+ cells per unit area in TBI tissues (n = 5; *p < 0.05; **p < 0.01; ***p < 0.001). (i) Immunofluorescence images showing α‐SMA (red) for vascular like structures, and CD44 (red) with Aggrecan (green) as markers of chondrocyte‐like cells. DAPI (blue) indicates nuclei. Scale bar: 100 µm. (j,k) Quantification of α‐SMA‐positive vessel‐/myofibroblast‐associated structures per unit area and chondrocyte‐like cells per unit area in TBI tissues (n = 5; *p < 0.05; **p < 0.01; ***p < 0.001). (l) Immunofluorescence staining for collagen I and III (both red), assessing extracellular matrix remodeling in TBI tissues. DAPI (blue) indicates nuclei; scale bar: 100 µm. (m–n) Quantification of Col I and Col III fluorescence intensity per unit area (n = 5; *p < 0.05; **p < 0.01; ***p < 0.001). Data are presented as mean ± SD. Quantitative comparisons in panels (d), (e), (g), (h), (j), (k), (m), and (n) were analyzed by one‐way ANOVA followed by Bonferroni's post hoc test. n = 5 animals per group. ns, not significant; *p < 0.05; **p < 0.01; ***p < 0.001; ****p < 0.0001.

To validate the in vivo omics results, we conducted histological analyses of one week post‐surgery in the rat MRCT model (Figure 9a,b). Immunofluorescence staining was performed to assess macrophage polarization. GO/Col scaffolds showed reduced iNOS+/CD68+ M1 macrophages and increased ARG1+/CD68+ M2 macrophages compared to MRCT and Col groups (Figure 9c). Quantitative analysis confirmed a statistically significant decrease in M1 macrophages and an increase in M2 polarization in the GO/Col group (Figure 9c–e), indicating an immunomodulatory effect that favors tissue repair. To evaluate regenerative activity, SOX9 and PCNA expressions were analyzed (Figure 9f). GO/Col scaffolds significantly increased the number of SOX9+ and PCNA+ cells, indicating enhanced stem cell proliferation and chondrogenic potential. Quantification revealed statistically higher SOX9+ and PCNA+ cell counts in the GO/Col group compared to all other groups (Figure 9f–h), confirming its stimulatory effect on tissue regeneration. Neovascularization and chondrogenic differentiation were assessed using α‐SMA (vascular marker), CD44, and Aggrecan (chondrogenic markers). GO/Col scaffolds markedly enhanced angiogenesis and increased the number of CD44+/Aggrecan+ cells within the repair site (Figure 9i). Statistical analysis confirmed these findings (Figure 9i–k), further supporting the regenerative advantage conferred by GO/Col scaffolds.

In addition, Immunofluorescence staining at 2 weeks post‐surgery revealed a marked increase in both Collagen I and Collagen III expression in the GO/Col group relative to all other groups (Figure 9l). Quantitative analysis showed significantly elevated fluorescence intensity of Col I and Col III in the GO/Col group, whereas MRCT and Col groups showed minimal or no significant increases compared to the control. These findings suggest that the GO/Col scaffold facilitates early and robust extracellular matrix remodeling, promoting a regenerative microenvironment conducive to functional TBI repair (Figure 9l–n).

These results collectively demonstrate that GO/Col scaffolds promote tendon‐to‐bone healing by reshaping the immune microenvironment, enhancing stem cell proliferation and differentiation, and stimulating angiogenesis and chondrogenic repair.

#### 2.3.6 GO/Col Scaffold Displays Promising In Vivo Biocompatibility

**FIGURE 10:**
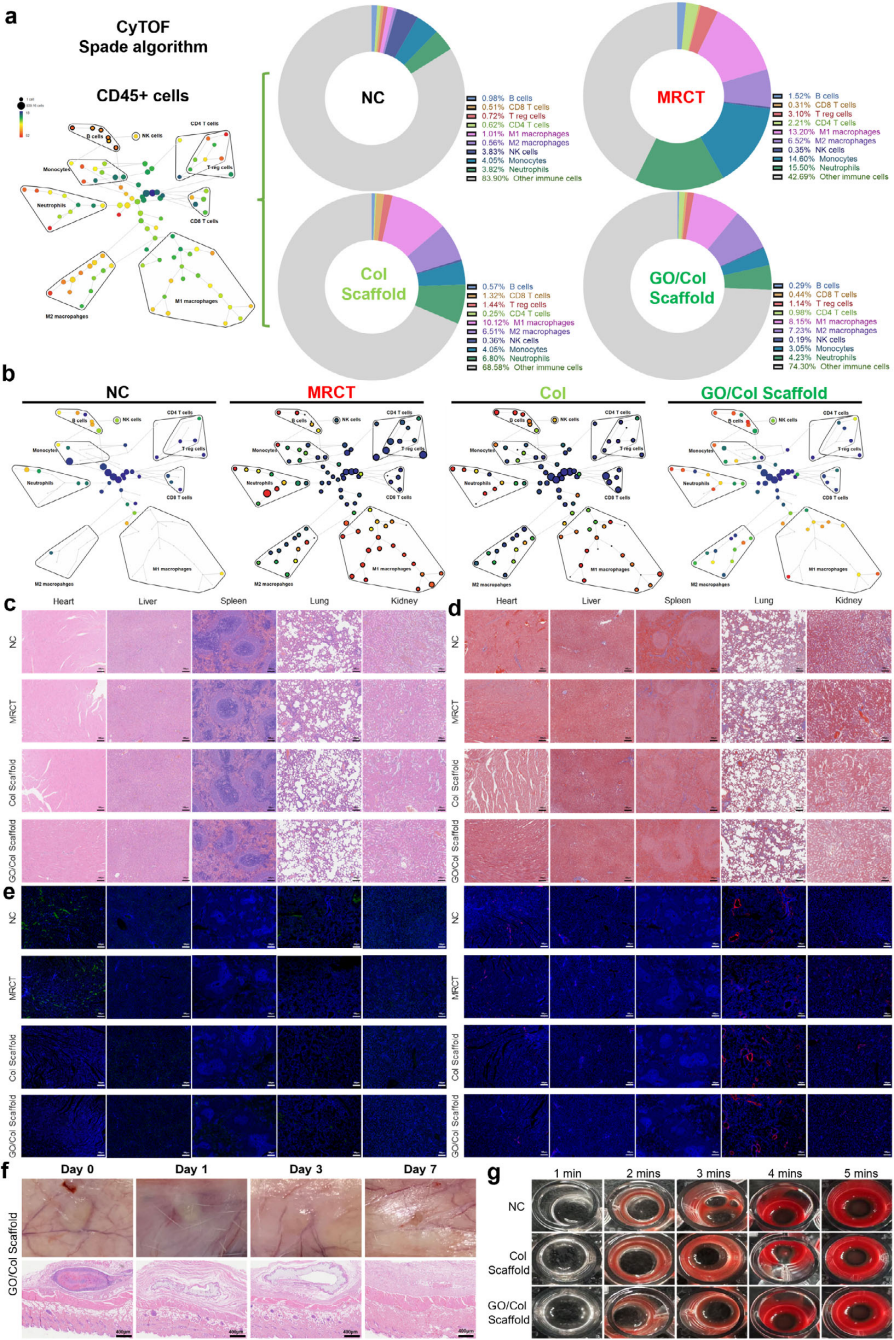
GO/Col scaffold exhibits favorable in vivo immunocompatibility. (a) CyTOF analysis of peripheral immune cells following scaffold implantation in a mouse MRCT model. Left: SPADE‐based clustering of immune cells; right: distribution of immune cell subtypes across groups. (b) SPADE tree visualization of group‐wise immune cell distribution. (c–d) H&E and Masson staining of major organs (heart, liver, spleen, lung, kidney) in each treatment group. Scale bar: 200 µm. (e) Immunofluorescence staining of F4/80 and α‐SMA in major organs to assess immune cell infiltration and fibrosis. Scale bar: 200 µm. (f) Gross appearance and H&E staining of subcutaneous scaffold implantation sites at different time points in rats. Scale bar: 400 µm. (g) In vitro coagulation assay using fresh rat whole blood to evaluate the procoagulant properties of the scaffold materials. Data are presented as mean ± SD where applicable. Quantitative comparisons of immune cell proportions in panels (a) and (b), as well as coagulation‐related measurements in panel (g), were analyzed by one‐way ANOVA followed by Bonferroni's post hoc test.

To evaluate systemic immune responses, CyTOF analysis combined with SPADE clustering was performed on peripheral blood samples from the rotator cuff injury model. The results demonstrated that mice in the GO/Col scaffold group exhibited reduced proportions and absolute numbers of neutrophils, macrophages, and monocytes compared to the MRCT and Col groups, suggesting a mitigated systemic immune response and immunomodulatory capacity of the scaffold (Figure 10a,b).

Histological assessments of major organs, including heart, liver, spleen, lung, and kidney—were conducted via H&E and Masson's trichrome staining to detect structural damage or fibrosis (Figure 10c,d). Across all groups, rats maintained normal activity, appetite, and neurological responses with no signs of systemic toxicity. Tissue sections from the GO/Col group revealed preserved organ architecture, intact cellular morphology, and no significant inflammatory cell infiltration or abnormal immune cell proliferation. Immunofluorescence staining for F4/80 and α‐SMA further confirmed the absence of fibrosis or immune infiltration, consistent with the histological findings (Figure 10e).

To assess local biocompatibility and degradation behavior, subcutaneous implantation of scaffolds was performed in rats, followed by macroscopic and histological evaluation from day 1 to day 7 post‐implantation (Figure 10f). All animals recovered uneventfully without signs of local infection, inflammation, or abnormal secretion. H&E staining showed no notable infiltration of inflammatory cells or abnormal proliferation of macrophages, mast cells, or lymphocytes. The gross scaffold architecture was largely resorbed by day 7 in the subcutaneous model, likely reflecting rapid degradation of the collagen‐dominant matrix rather than definitive complete elimination of the GO component. Finally, a coagulation assay using fresh rat blood was conducted to examine hemocompatibility. The GO/Col scaffold did not significantly alter clotting time, further supporting its blood compatibility (Figure 10g).

Collectively, these findings demonstrate that the GO/Col scaffold is safe, non‐toxic, and exhibits excellent in vivo and hemocompatibility, making it a promising candidate for clinical translation.

## 3 Discussion

The present study systematically characterized the physicochemical properties, biological performance, and regenerative potential of a graphene oxide/collagen (GO/Col) hybrid scaffold in the context of chronic massive rotator cuff tear (MRCT) repair. By combining wet incubation with freeze‐drying, we generated a porous GO/Col scaffold with preserved three‐dimensional architecture, favorable wettability, improved compressive stability, and good cytocompatibility. Integrated in vitro, in vivo, histological, biomechanical, and multi‐omics analyses further suggested that this scaffold may improve tendon–bone healing through coordinated effects on matrix remodeling, mesenchymal lineage bias, and immune microenvironment modulation. Overall, these findings support the translational potential of GO/Col scaffolds as a bioactive strategy for chronic MRCT repair.

Wet incubation allowed GO nanosheets to infiltrate collagen interstices uniformly, while freeze‑drying solidified this dispersion into a stable hybrid network whose porosity (70–85 %), pore interconnectivity, and wall roughness could be predictably tuned by the GO fraction. Traditional air‑drying, by contrast, induced capillary collapse, GO restacking, and irregular micro‑voids that compromise both cell ingress and mechanical isotropy. XPS and EDS verified that the hybrid remained chemically pure, containing only C, N, and O, with an increasing C/O ratio proportional to GO content—consistent with a non‑covalent but intimate GO–collagen association. Raman and Fourier‑transform infrared spectra further substantiated the presence of characteristic D/G (GO) and Amide (collagen) bands, underscoring the structural integrity of both components after processing. Importantly, no exogenous cross‑linkers were required, circumventing concerns over cytotoxic residues and preserving pro‑regenerative bioactivity [35, 36].

A key prerequisite for implantable scaffolds is a degradation rate that matches the tempo of native tissue remodeling [37, 38]. Immersion in phosphate‑buffered saline (37°C, pH 7.4) showed that mass loss slowed progressively with greater GO loading, reflecting enhanced matrix cohesion and resistance to enzymatic and hydrolytic attack. Parallel measurements of cumulative GO elution revealed a proportional, sustained release profile—rapid during the first 48 h, then tapering to a plateau by Day 21—indicating that GO acted as both a reinforcement and a long‑acting bioactive cue. Such temporal alignment is advantageous for MRCT, where early mechanical stability must be coupled to weeks‑long molecular signaling for optimal TBI maturation [39, 40, 41].

Mechanical characterization indicated that the addition of GO did not lead to a generalized improvement in tensile performance. In our system, the GO‐containing scaffolds showed lower ultimate tensile strength and lower Young's modulus than the collagen‐only scaffold, suggesting that GO incorporation changed the tensile mechanics of the collagen matrix and may have increased material heterogeneity. By contrast, the GO‐containing groups exhibited greater compressive resistance, especially in the high‐strain region, implying that GO contributed more strongly to compression‐bearing structural support than to tensile reinforcement. This distinction is important for interpreting the scaffold's role at the tendon–bone interface, where maintenance of local interfacial architecture under complex loading may be as relevant as pure tensile strength. Moreover, all GO/Col scaffolds retained relatively good hydrophilicity, which is favorable for cellular attachment and integration.

SEM revealed a gradual transition from circular, ∼200 µm pores in the collagen control to ellipsoidal or slit‑like pores at higher GO ratios; this increased tortuosity augmented specific surface area and provided topographical guidance for cell elongation. Fewer but more interconnected pores—especially in the GO‑0.6 composition—facilitated deeper cellular infiltration and angiogenic sprouting, while smaller pore throats conferred additional resistance to premature degradation. These observations align with prior findings that hierarchical porosity (50‑250 µm) maximizes vascular ingrowth and osteochondral interface formation [42, 43, 44].

BMSCs seeded on GO/Col scaffolds displayed accelerated adhesion within 2 h, enhanced proliferation (1.8‑fold DNA content by Day 5), and extended lamellipodia—attributes linked to GO's ability to enrich FAK and PI3K/Akt signaling  [45, 46]. Osteogenic, chondrogenic, and adipogenic differentiation assays corroborated a lineage‑selective bias: alkaline‑phosphatase activity, mineralized nodule formation, and glycosaminoglycan deposition increased significantly, whereas Oil‑Red‑O‑positive lipid vacuoles decreased. Concomitant qPCR confirmed up‑regulation of Sfrp2, Tent5c, Gbx2, Gdf6, and Erg, with suppression of Ppargc1a, Adipoq, and Cidec. These molecular signatures suggest that GO/Col matrices tip the balance toward bone‑ and fibrocartilage‑forming phenotypes while deterring fatty infiltration—an outcome highly desirable in chronic MRCT where myosteatosis thwarts healing [47, 48]. Although BMSCs were used here as a tractable mesenchymal progenitor model to examine scaffold‐associated adipogenic suppression, FAPs are the principal stromal population driving fibro‐fatty degeneration in chronically injured rotator cuff muscle. Therefore, the present in vitro findings should be interpreted as evidence that GO/Col can suppress adipogenic potential in a mesenchymal context, rather than as direct proof of FAP‐specific regulation in vivo.

CyTOF profiling and in vitro polarization assays revealed that GO/Col scaffolds curtailed pro‑inflammatory M1 markers (IL‑6, Nlrp3) and boosted anti‑inflammatory M2 cytokines (IL‑10, TGF‑β, ApoE). Transcriptomic data supported increased Bcl‑6 and ghrelin expression, both implicated in M2 skewing [49, 50]. Fluorescence staining of explanted tissues evidenced a three‑fold rise in CD206^+^ (M2) versus CD86^+^ (M1) cells at Day 14. By attenuating early inflammatory signaling and creating a more reparative microenvironment, the scaffold may support the survival and functional integration of reparative cells at the healing interface. Our in vivo immune profiling mainly captured an early postoperative time window and therefore should be interpreted as a snapshot of the evolving immune response rather than a full description of the temporal dynamics of macrophage‐state transitions. Because the switch from acute inflammation to reparative remodeling is highly time dependent during enthesis healing, future longitudinal profiling will be necessary to define the duration, sequence, and functional significance of these immune shifts.

It should be noted that the M1/M2 classification used in the present study represents a simplified heuristic framework for describing macrophage‐associated inflammatory versus reparative features, rather than a strict binary model of in vivo macrophage states. In the healing tendon–bone interface, macrophages exist along a dynamic and heterogeneous continuum shaped by local mechanical, matrix, and cytokine cues. Therefore, the changes in CD86/iNOS‐ and CD163/ARG1‐associated signals observed here should be interpreted more cautiously as indicating a shift toward a more reparative, anti‐inflammatory macrophage‐associated phenotype, rather than a complete or discrete conversion from “M1” to “M2.” This distinction is particularly important in the context of tissue repair, where macrophage plasticity is temporally regulated and functionally context dependent. In addition, RAW264.7 cells are a transformed murine macrophage‐like cell line and may not fully recapitulate the phagocytic behavior, activation thresholds, and polarization plasticity of primary macrophages in vivo. Accordingly, the present in vitro data should be interpreted as model‐system evidence of scaffold‐associated macrophage phenotypic bias, rather than definitive proof of primary macrophage reprogramming.

Building upon these in‑vitro insights, the GO/Col scaffold was implanted into a rat chronic MRCT model that replicates key human pathologies (muscle retraction, fatty infiltration, tendon fibrosis). CatWalk gait analysis showed earlier restoration of stride length and paw‑pressure symmetry, while forelimb grip strength rose to 85 % of naïve values by Week 8—double that of collagen controls. Ex vivo mechanical pullout tests demonstrated 140 % greater maximum load‑to‑failure and 120 % higher stiffness relative to MRCT repairs without scaffolds. Micro‑computed tomography revealed BMD at the tendon footprint recovered to 90 % of normal, accompanied by organized Sharpey‑like collagen fibers and fibrocartilage regeneration, as evidenced by Masson, Safranin‑O/fast green, and aggrecan immunostaining.

To unravel the orchestrated events underpinning these outcomes, integrated mRNA‑seq, inflammation arrays, and CyTOF analyses were conducted. Gene Ontology and GSEA highlighted enriched Wnt/β‑catenin, cytoskeletal re‑arrangement, and ECM‑receptor pathways in BMSCs; angiogenic Vegfα and matrix‑remodeling Mmp13 transcripts were also elevated. Complementary protein arrays confirmed reductions in TNF‑α, IL‑1β, and MPO, corroborating a suppressed neutrophil/oxidative burst. Together, these datasets portray a coordinated modulation of lineage specification, vasculogenesis, and immune quiescence orchestrated by the GO/Col interface [11, 15, 51].

A variety of biomaterials have been explored for rotator cuff repair, but their regenerative efficacy remains limited by poor integration, rapid resorption, or insufficient biological activity [52, 53, 54]. The present GO/Col construct surpasses many predecessors by coupling nanoscale reinforcement with biological signals endemic to the native ECM. Similar trends were reported by Su et al. [55] using GO‑doped PLGA membranes; however, those lacked the collagenous motifs essential for tenogenic cues and demonstrated inferior hydrophilicity. Because α‐SMA is expressed not only by vascular smooth muscle cells but also by myofibroblasts, the present staining should be interpreted cautiously and not as definitive endothelial evidence of neovascularization, even though quantification was restricted to morphologically organized vessel‐like structures. Our study uniquely integrates multi‑omics evidence, chronic pathology modeling, and functional gait metrics to build a holistic performance profile.

Clinically, MRCTs exhibit failure rates > 40 % with current suture‑anchor techniques, chiefly because scar‑mediated entheses cannot replicate the graded fibrocartilage‑bone continuum. By mechanically reinforcing the repair site, directing progenitor differentiation, and tempering inflammation, the GO/Col scaffold offers a multifaceted solution that could reduce re‑tear incidence and accelerate rehabilitation timelines. Going forward, integrating controlled‑release biologics (e.g., BMP‑7, IL‑4) or aligning fibers via 3D bioprinting may further potentiate functionality. Moreover, the scaffold's immunomodulatory prowess suggests applications beyond orthopedics—such as myocardial patching or chronic wound dressings—where balanced inflammation resolution is pivotal.

Several limitations should be acknowledged. First, BMSCs were used as a mesenchymal progenitor model and therefore do not fully recapitulate the biology of fibro‐adipogenic progenitors involved in chronic MRCT‐associated fibro‐fatty degeneration. Second, the macrophage experiments were performed using RAW264.7 cells, and the observed changes in inflammatory and reparative marker profiles should therefore be interpreted as model‐system evidence rather than definitive proof of primary macrophage reprogramming. Third, the M1/M2 framework used here represents a simplified description of macrophage‐associated states and does not fully capture their in vivo heterogeneity. Fourth, the rapid disappearance of the scaffold in the subcutaneous model likely reflects degradation of the collagen‐dominant matrix rather than complete clearance of the GO component, whose long‐term fate remains to be clarified. Finally, because α‐SMA is not an endothelial‐specific marker, the corresponding findings should be interpreted cautiously as vascular‐/myofibroblast‐associated remodeling rather than definitive neovascularization. Despite these limitations, the overall in vitro, in vivo, histological, biomechanical, and multi‐omics data consistently support the beneficial effects of the GO/Col scaffold on the reparative microenvironment and tendon–bone healing in chronic MRCT.

### 3.1 Conclusion

In summary, the GO/Col hybrid scaffold fabricated by a wet‐incubation/freeze‐drying strategy provided a porous and biocompatible collagen‐based platform with GO‐associated bioactivity and improved compressive stability. Across in vitro assays and a chronic MRCT model, the scaffold was associated with enhanced mesenchymal cell activity, favorable immune modulation, reduced muscle degeneration, improved tendon–bone regeneration, and better functional recovery. Multi‐omics analyses further suggested that these effects may involve coordinated regulation of immune, lineage‐related, and matrix‐remodeling pathways. Collectively, these findings support the translational potential of GO‐based collagen scaffolds for musculoskeletal repair and provide mechanistic clues that may inform the design of next‐generation bioactive scaffolds for tendon–bone healing.

## 4 Materials and Methods

### 4.1 Preparation of Type I Collagen From Porcine Dermis

Porcine dermis was harvested from freshly obtained skin and trimmed to remove residual fat and hair. The tissue was washed in absolute ethanol for 24 h to remove lipids and then decalcified in 0.5 M EDTA (pH 7.4) at 4°C with gentle stirring for 48 h. The decalcified dermis was digested with pepsin (1 mg/mL in 0.5 M acetic acid) at 4°C for 48–72 h. The solution was centrifuged (10 000×g, 30 min, 4°C) and the supernatant was dialyzed against deionized water for 72 h with daily water changes. The dialysate was lyophilized and reconstituted in 0.5 M acetic acid to a final collagen concentration of 1%–2% (w/v). The solution was stored at 4°C until use.

### 4.2 Fabrication of Collagen (Col) and Graphene Oxide/Collagen (GO/Col) Scaffolds

To fabricate porous collagen scaffolds, 1% collagen solution was poured into Teflon molds (25 × 25 × 2 mm) and frozen at –80°C for 12 h. The frozen gels were freeze‐dried (48 h, –55°C) to obtain sponge‐like porous matrices. The scaffolds were cross‐linked by immersion in 50 mM EDC and 20 mM NHS in 95% ethanol at room temperature for 12 h. After cross‐linking, scaffolds were washed with sterile PBS and lyophilized again. These served as the Col control group.

For GO/Col scaffolds, monolayer graphene oxide (GO, purchased from XFNANO, Nanjing, China) was dispersed in ultrapure water at concentrations of 0.2, 0.6, or 1.0 mg/mL (Equal to Col‐0.1 = Col‐L; Col‐0.6 = Col‐M; Col‐1.0 = Col‐H). GO solution (800 µL) was pipetted uniformly onto half‐sized Col disks (12.5 × 12.5 × 2 mm) and immediately frozen at –80°C, followed by freeze‐drying for 12 h. Air‐dried GO/Col scaffolds (left at room temperature in a fume hood for 12 h) were used as a comparative processing control. All scaffolds were UV‐sterilized (30 min per side) and stored at 4°C in sterile containers. Col = Col Scaffold; Col‐0.1 = Col‐L; Col‐0.6 = Col‐M; Col‐1.0 = Col‐H. Air‐dried scaffolds were prepared as a processing‐control group for preliminary comparison. Only scaffolds that maintained acceptable morphological integrity and physicochemical suitability after initial characterization were advanced to subsequent biological experiments. Based on this screening, the air‐dried scaffolds were excluded from further in vitro and in vivo studies.

### 4.3 Morphological and Physicochemical Characterization

#### 4.3.1 Scanning Electron Microscopy

Scaffold samples were sputter‐coated with platinum and imaged using a Hitachi, SU8200 SEM at an accelerating voltage of 5 kV. Pore diameter and surface roughness were quantified using ImageJ (NIH, USA) from 5 random fields per scaffold.

#### 4.3.2 Raman Spectroscopy

Raman spectra were recorded using a 514 nm laser excitation (HORIBA Jobin Yvon HR800). Spectra were collected over 750–2250 cm^−^^1^.

#### 4.3.3 Fourier‐Transform Infrared Spectroscopy

Spectra were obtained using a Tensor 27, Bruker, Germany FTIR spectrometer in the range 4000–400 cm^−^^1^.

#### 4.3.4 X‐ray Photoelectron Spectroscopy

XPS spectra (Thermo Scientific K‐Alpha, USA) were acquired using a monochromatic Al Kα source. High‐resolution scans of C 1s and O 1s were analyzed for functional group content.

#### 4.3.5 Water Contact Angle Measurement

Static contact angles were measured using a 2 µL droplet of ultrapure water on a goniometer (Model SL200B, Solon, China). Data from 5 different locations per sample were averaged.

#### 4.3.6 Thermogravimetric Analysis with Mass Spectrometry

TG‐MS was conducted under Ar flow from room temperature to 800°C at 10°C/min (TA instruments, SDT 650‐Discovery MS) to evaluate scaffold decomposition and gas evolution.

#### 4.3.7 Surface Elemental Composition (EDS and Mapping)

Elemental composition and distribution on scaffold surfaces were analyzed using energy‐dispersive X‐ray spectroscopy (EDS) attached to a scanning electron microscope (Hitachi, SU8200). Samples were mounted on aluminum stubs, sputter‐coated with a thin layer of platinum and imaged at 5 kV. EDS spectra were recorded at three randomly selected areas per sample. Elemental mapping (C, O, N for collagen and C, O for GO) was performed in scanning mode to visualize the homogeneous or heterogeneous distribution of GO on the scaffold surface.

### 4.4 In Vitro Degradation and GO Release

Dry scaffolds (∼10 mm^2^) were immersed in 10 mL PBS (pH 7.4) at 37°C under static conditions. At specified time points (Days 1, 7, 14), scaffolds were removed, blotted, dried, and weighed. Degradation (%) was calculated as:

$$\mathrm{Mass}\;\mathrm{loss}=\left(W_{o}-W_{t}\right)/W_{o}\times 100\%$$

For GO release, scaffold supernatants were collected at 12 h, 1, 3, 7, and 14 days. GO concentration was quantified by absorbance at 230 nm (Thermo Multiskan Go), based on a standard curve. Cumulative release was expressed as a percentage of total GO loaded.

### 4.5 Mechanical Property Testing

#### 4.5.1 Tensile Test

Rectangular samples (60 × 10 mm) were tested using a universal testing machine (CMT‐4202, MTS, China) with a 50 N load cell. A 0.15 N preload was applied, and samples were extended at 5 mm/min. Young's modulus and ultimate tensile strength were calculated from stress–strain curves.

#### 4.5.2 Compression Test

Cylindrical samples (12 × 12 × 2 mm) were compressed at 5 mm/min until 40% strain. Compressive modulus was derived from the slope of the stress–strain curve between 10% and 20% strain.

### 4.6 In Vitro Cell Culture and Bioassays

#### 4.6.1 Cell Culture

Human bone marrow‐derived mesenchymal stromal cells (BMSCs; Bone Marrow–Derived Mesenchymal Stem Cells, Normal, Human; ATCC, Manassas, VA, USA; Cat. No. PCS‐500‐012, RRID: N/A) and murine RAW 264.7 macrophages (ATCC, TIB‐71, RRID: CVCL_0493) were cultured in mesenchymal stem cell basal medium supplemented with 10% fetal bovine serum (FBS) and 1% penicillin–streptomycin, and in high‐glucose Dulbecco's modified Eagle's medium (DMEM) supplemented with 10% FBS and 1% penicillin–streptomycin, respectively. Cells at passages ≤3 were used for all experiments. All cultures were routinely tested for mycoplasma contamination using a commercial PCR‐based kit and were confirmed to be mycoplasma‐free throughout the study.

#### 4.6.2 Cell Proliferation CCK‐8 Assay

BMSC viability was assessed using the Cell Counting Kit‐8 (CCK‐8; Beyotime, China). BMSCs were seeded in 96‐well plates (Corning, USA) at a density of 1 × 10^3^ cells per well and cultured with the indicated scaffold‐derived treatments for 24 h. Subsequently, 10 µL of CCK‐8 reagent was added to each well, followed by incubation for 2 h at 37°C. Absorbance at 450 nm was measured using a microplate reader (Bio‐Rad, USA). Cell viability was expressed as the percentage of the mean optical density in each treatment group relative to that of the control group.

#### 4.6.3 BrdU assay

BMSC proliferation was further evaluated using a 5‐bromo‐2′‐deoxyuridine (BrdU) Assay Kit (Cell Signaling Technology, USA) according to the manufacturer's instructions. BMSCs were seeded in 6‐well plates at a density of 1 × 10^6^ cells per well and exposed to the indicated treatments for 24 h. BrdU solution was added during the final 12 h of treatment. Cells were then fixed, washed twice with phosphate‐buffered saline (PBS), and incubated with a rat anti‐BrdU primary antibody at room temperature for 1 h. Nuclei were counterstained with DAPI for 5 min. Cell proliferation was quantified as the percentage of BrdU‐positive cells relative to total DAPI‐positive cells, or as the number of BrdU‐positive cells per mm^2^.

#### 4.6.4 Live/Dead Cell Staining

BMSC viability after exposure to scaffold‐derived extracts was evaluated using a live/dead cell staining kit (Beyotime, China). BMSCs were seeded in 24‐well plates at a density of 5 × 10^4^ cells per well and cultured for 24 h. The culture medium was then replaced with the corresponding scaffold extract, and incubation was continued for another 24 h. Cells were washed twice with phosphate‐buffered saline (PBS) and incubated with 300 µL of staining solution containing calcein AM and propidium iodide (PI) at 37°C for 30 min in the dark. Live cells were identified by green fluorescence and dead cells by red fluorescence. Fluorescence images were acquired using a confocal microscope (Nikon, Japan).

#### 4.6.5 Migration Assay

A scratch wound‐healing assay was performed on confluent BMSC monolayers. After scratching, cells were cultured with the indicated scaffold‐derived treatments, and wound closure was imaged at 12 and 24 h using phase‐contrast microscopy. The migration rate was quantified as the percentage of wound closure using ImageJ.

#### 4.6.6 Multilineage Differentiation

BMSCs seeded on the scaffolds were subjected to osteogenic, adipogenic, or chondrogenic induction for 21 days. For all assays, BMSCs were plated in 6‐well plates at a density of 1 × 10^5^ cells per well and allowed to attach for 48 h before induction. Osteogenic medium consisted of basal osteogenic medium supplemented with ascorbic acid, glutamine, β‐glycerophosphate, penicillin–streptomycin, fetal bovine serum, and dexamethasone. Adipogenic medium consisted of basal adipogenic medium supplemented with dexamethasone, penicillin–streptomycin, rosiglitazone, glutamine, IBMX, insulin, and fetal bovine serum. Chondrogenic medium consisted of basal chondrogenic medium supplemented with ascorbic acid, proline, penicillin–streptomycin, ITS, sodium pyruvate, fetal bovine serum, dexamethasone, and TGF‐β1. The induction media were refreshed every 2–3 days for osteogenesis, every 2 days for adipogenesis, and every 3 days for chondrogenesis.

After 21 days of induction, cells were washed with PBS, fixed in 4% paraformaldehyde for 30 min at room temperature, and stained with lineage‐specific dyes. Osteogenic differentiation was assessed by Alizarin Red staining for calcium deposition, adipogenic differentiation by Oil Red O staining for lipid accumulation, and chondrogenic differentiation by Safranin O staining for proteoglycan‐rich matrix formation. Stained cultures were imaged under a light microscope. In parallel, lineage‐related gene expression was evaluated by RT‐qPCR using SYBR Green (Bio‐Rad), and the primer sequences are listed in Table S2.

#### 4.6.7 Macrophage Polarization

RAW264.7 cells were seeded in 6‐well plates at a density of 1 × 10^6^ cells per well and cultured for 24 h. The medium was then replaced with scaffold‐derived extract supplemented with lipopolysaccharide (LPS, 1000 ng/mL), and cells were further incubated for 24 h. Macrophage‐associated inflammatory phenotypes were evaluated by immunofluorescence staining for F4/80, CD163, and inducible nitric oxide synthase (iNOS). Flow cytometry was performed as a complementary method to assess the expression of macrophage‐associated markers under different scaffold conditions.

#### 4.6.8 Flow Cytometry Analysis (FACS)

Single‐cell suspensions were stained with monoclonal antibodies (eBioscience) for BMSC (CD73^+^, CD90^+^, CD105^+^, CD34^−^, CD45^−^, HLA‐DR^−^) and macrophage (CD86, F4/80, CD163) markers. Data were acquired using a Beckman Coulter FC‐500.

##### 4.6.8.1 Apoptosis assay

Apoptosis was assessed utilizing the Annexin V‐FITC Apoptosis Detection Kit (Beyotime, China), a trusted methodology [56]. BMSCs were seeded in 6‐well plates at a density of 1 × 10^6^ cells per well and cultured for 24 h. The medium was then replaced with the corresponding scaffold extract for 6 h. Cells were harvested by trypsinization, washed twice with PBS, and resuspended in Annexin V binding buffer. Annexin V‐FITC and propidium iodide (PI) were added according to the manufacturer's instructions, and the cells were incubated for 15 min at room temperature in the dark. Samples were analyzed by flow cytometry (BD, USA) to determine apoptotic cell populations.

##### 4.6.8.2 Cell Cycle Analysis

To evaluate the effect of scaffolds on BMSC proliferation at the cell cycle level, flow cytometry‐based cell cycle analysis was performed. BMSCs were seeded onto scaffolds in 24‐well plates (1 × 10^5^ cells/well) and cultured for 48 h. Cells were then harvested by trypsinization, washed twice with cold PBS, and fixed in 70% ice‐cold ethanol overnight at –20°C. Fixed cells were washed with PBS and incubated with propidium iodide (PI) staining buffer (50 µg/mL PI, 100 µg/mL RNase A in PBS) in the dark at room temperature for 30 min. Samples were analyzed using a flow cytometer (e.g., Beckman Coulter FC‐500 or BD FACSCalibur). A total of at least 10 000 events were recorded per sample. Cell cycle distribution was determined using FlowJo (v10.0) software by quantifying the percentage of cells in G0/G1, S, and G2/M phases based on DNA content. The proportion of cells in S phase was used as an index of proliferation activity. G2/M phase distribution was also compared to evaluate mitotic activity under different scaffold conditions.

#### 4.6.9 Cell Adhesion Assay

BMSCs were seeded onto sterilized scaffolds (1 × 10^4^ cells/scaffold) in 24‐well plates and cultured under standard conditions (37°C, 5% CO_2_). After 1, 4, and 24 h, scaffolds were gently washed with PBS to remove non‐adherent cells. Adherent cells were fixed with 4% paraformaldehyde, permeabilized with 0.1% Triton X‐100, and stained with Alexa Fluor 488‐phalloidin (for F‐actin) and DAPI (for nuclei). Cytoskeletal spreading and morphology were observed using a fluorescence microscope (Leica DMi8). Cell adhesion efficiency was quantified by counting the number of adherent cells per scaffold from fluorescence images using ImageJ. Data were expressed as cells/mm^2^ and compared among groups.

#### 4.6.10 Detection of Intracellular Reactive Oxygen Species (ROS)

To assess oxidative stress levels in BMSCs cultured on different scaffolds, intracellular ROS generation was detected via immunofluorescence staining using a ROS‐sensitive fluorescent probe. BMSCs were seeded onto Col, GO‐L/Col, GO‐M/Col, and GO‐H/Col scaffolds (1 × 10^5^ cells/scaffold) and co‐cultured for 24 h under standard culture conditions (37°C, 5% CO_2_, humidified atmosphere). After 24 h of incubation, scaffolds with adherent cells were gently washed three times with warm PBS to remove non‐adherent cells and culture medium. Intracellular ROS was then labeled using a ROS assay kit (e.g., ROS Assay Kit, DCFH‐DA probe, Beyotime or equivalent). Scaffolds were incubated with 10 µM DCFH‐DA working solution diluted in serum‐free medium for 30 min at 37°C in the dark. After incubation, cells were washed thoroughly with PBS to remove excess unhydrolyzed probe. Following ROS probe staining, cells were fixed in 4% paraformaldehyde for 15 min at room temperature. Nuclei were counterstained with DAPI (1 µg/mL, 5 min), and scaffolds were mounted with anti‐fade mounting medium. Samples were imaged immediately using a fluorescence microscope (Leica DMi8 or equivalent) with appropriate filter sets for DCF (excitation 488 nm, emission 525 nm) and DAPI. Fluorescence intensity representing intracellular ROS levels was quantified from at least five randomly selected fields per sample using ImageJ software. ROS levels were normalized to the number of nuclei to ensure comparability.

### 4.7 Real‐Time Quantitative Polymerase Chain Reaction

To evaluate the abundance of mRNA under the cellular level, total RNA was meticulously extracted from the cells and muscles employing the Trizol reagent (Invitrogen) and subsequently quantified using the Nanodrop instrument (Thermo Scientific, USA) [57]. Subsequently, to gauge mRNA expression levels, cDNA served as a template in qPCR assays utilizing the TB GreenTM Premix Ex TaqTM II kit (Takara; RR820A), with GAPDH serving as an internal reference. Furthermore, qPCR primers designed for mRNA amplification were synthesized by Bioengineering (Shanghai, China). The relative expression levels of each sample were determined utilizing the comparative Ct method (2‐ΔΔCt), with a minimum of three independent replicates conducted. Finally, all values were normalized concerning the control condition. Primer sequences are listed in Table S2.

### 4.8 Animal Experiments

#### 4.8.1 Ethics Approval

All animal experimental protocols were approved by the Department of Laboratory Animal Science, Fudan University (2024‐HSYY‐597).

#### 4.8.2 Sample Size Calculation for Animal Experiments

In exploratory animal studies where accurate estimates of standard deviation and effect size are difficult to obtain in advance, the *resource equation method* is commonly used to estimate appropriate sample size. This method is particularly suitable for quantitative outcomes analyzed by ANOVA, and helps balance statistical power with ethical use of animals.

The equation is defined as:

$$E=N-K=\mathrm{Kn}-K=K\left(n-1\right)$$

Where:

- *E* is the degrees of freedom of the analysis (typically between 10 and 20),
- *N* is the total number of animals,
- *K* is the number of experimental groups,
- *n* is the number of animals per group.

The formula can be rearranged to calculate sample size per group: n = E/K + 1

In this study, *K = 4* groups were designed. Setting *E* between 10 and 20, the calculated *n* falls between 4 and 6 animals per group.

Considering two observation time points (4 and 8 weeks post‐operation), and the need for two biological replicates per group per time point (one for behavioral and biomechanical testing, and the other for imaging and histological analysis), the final required number of rats per group is 16–24.

Additionally, 10 extra rats were used for validating the chronic massive rotator cuff tear (MRCT) model. Therefore, the total number of rats required was estimated to be between 74 and 106.

Mice were only used for mechanistic studies and biocompatibility evaluation. Sample size was determined based on group‐specific omics requirements:

- RNA‐seq: 3 vs. 3
- CyTOF: 3 vs. 3
- Inflammatory cytokine array: 2 vs. 2
- Tissue flow cytometry validation: 3 vs. 3

Each test required a minimum of 11 mice per group, accounting for technical replicates and tissue needs. To address possible perioperative mortality, the per‐group sample size at each observation time point was increased to n+1 = 12, ensuring robustness of data even in case of accidental loss.

As a result, a total of 48 mice were used in this study.

#### 4.8.3 Construction and Repair of the Chronic MRCT Model in Rats

A chronic massive rotator cuff tear (MRCT) model was established in rats and subsequently repaired using different scaffold interventions. Rats were randomly divided into four groups: sham‐operated group (NC), MRCT repair with suture only (MRCT), MRCT repair with suture plus collagen I scaffold (Col), and MRCT repair with suture plus GO/Col scaffold (GO/Col). After anesthetizing rats via intraperitoneal injection of 5% pentobarbital sodium (45 mg/kg), the surgical area around the shoulder was shaved and disinfected. A longitudinal incision was made along the humeral axis to expose the supraspinatus tendon. The tendon was sharply detached from the greater tuberosity, and the bony footprint was debrided. To simulate chronic tendon retraction and degeneration, a collagen membrane was sutured over the tendon stump to prevent reattachment, and the wound was closed in layers. After a 12‐week degeneration period, the supraspinatus tendon was surgically repaired. Bone tunnels were created through the greater tuberosity using Kirschner wires, and the retracted tendon was reattached using a modified Mason–Allen technique. In the Col and GO/Col groups, scaffolds were sutured to the tendon‐bone interface before fixation. Finally, the joint capsule, deltoid, and skin were sutured sequentially, and animals were returned to labeled cages. The NC group received exposure of the supraspinatus only without further intervention, while the MRCT group underwent surgical repair without scaffold implantation. Animals were allowed free cage activity after surgery and that no external immobilization was used.

The rat model was defined as a chronic massive rotator cuff tear not by absolute tear size, but by the combination of:

- complete supraspinatus tendon detachment,
- a prolonged chronic degeneration window,
- tendon retraction, and
- established muscle degeneration characterized by fatty infiltration and fibrosis before repair.

#### 4.8.4 Micro‐Computed Tomography

Samples were scanned using a micro‐CT system at 35 µm resolution, with 65 kV voltage, 378 µA current, and a 1.0 mm aluminum filter. After accurate positioning, scans were performed, and data were reconstructed using NRecon software. Quantitative analysis was carried out with CTAn and CTvox software to evaluate bone parameters, including bone mineral density (BMD) and bone volume fraction (BV/TV, %), specifically at the greater tuberosity of the humerus.

#### 4.8.5 HE Staining

Remove the TBI tissue and skeletal muscle tissue near the wound fixed in 4% paraformaldehyde, paraffin‐embedded the skeletal muscle tissue, cut it into thin slices and attach it to slides, xylene immersion for 10 min, 100%–90%–80%–70% ethanol gradient immersion for 5 min each time, rinsed in water for 5 min, hematoxylin staining for 5 min, rinsed in water, 5% acetic acid immersion for 1 min, rinsed in water, and Eosin staining for 1 min, water rinse, 70%–80%–90%–100% ethanol gradient immersion, 10 sec each time, xylene immersion for 1 min, natural drying, neutral gum sealing, observation and photographing under pathology microscope. Semi quantitative analysis: sections were observed under an optical microscope and photographed. The staining results were evaluated using the rotator cuff tendon bone healing histological scoring system (Table S4).

#### 4.8.6 Masson and Safranin O–Fast Green Staining

Masson: Remove the TBI and skeletal muscle tissue near the wound fixed in Bouin's solution, paraffin‐embedded the skeletal muscle tissue, cut it into thin slices and attached it to slides, deparaffinize the slices, oxidize the slices with 1% potassium permanganate for 5 min, bleach the slices with oxalic acid for 1 min after distilled water washing, stain with azurite blue for 5 min after distilled water washing, shake off the residual liquid after distilled water washing, drop stain with Mayer's hematoxylin for 3–5 min After washing with distilled water, the slices were stained with Mayer's hematoxylin for 3–10 min, rinsed with running water for 5–10 min, stained with Lichun red picric acid saturated solution for 5 min, washed with 1% aqueous acetic acid, sliced with 1% phosphomolybdic acid for 5 min, washed with distilled water, drop‐stained with 1% light green or toluidine blue for 30s, washed with 1% aqueous acetic acid, sliced with 95% alcohol, dehydrated with anhydrous ethanol, made transparent by xylene, sealed with neutral gum, and put under the observation of an inverted microscope.

Safranin O–Fast Green Staining: After deparaffinization and rehydration (as in HE staining), tissue sections were stained with fast green for 5 min and briefly washed with a weak acid solution. Sections were then stained with safranin O for 5 min at room temperature. Dehydration and mounting followed the same procedure as HE staining.

#### 4.8.7 Immunohistologic Analysis

Sections of the TBI tissue and skeletal muscle tissue were obtained by cutting samples into 5 µm thick slices, as previously described. The sections were subsequently subjected to dewaxing and rehydration using a gradient of xylene/ethanol and PBS. The surrounding water was meticulously cleared away, and each sheet of tissue was adorned with perfectly drawn circles using a highlighter. The Achilles tendon sections were then immersed in a solution consisting of 5% BSA and 0.5% Triton‐X‐100 (Servicebio, China) for 1 h at ambient temperature. Following this, they were incubated overnight at 4°C with the primary antibody. Subsequently, thorough washing with 1 × TBST was performed three times for a duration of 5 min each, both before and after an additional hour of incubation at room temperature with Alexa Fluor 488 anti‐rat (H + L) or Alexa Fluor 594 anti‐rat (H + L) secondary antibody (1:500; Abcam, USA). Finally, the visual documentation was accomplished through the employment of an immunofluorescence microscope (Leica, Germany).

For cellular immunofluorescence staining, RAW macrophages or BMSCs were seeded onto well plates and cultured in high‐sugar DMEM until attaining a confluence ranging from 30% to 60%. Then, diverse intervention methods were employed. Subsequently, the cells were delicately rinsed with PBS and fixed using 4% paraformaldehyde for 10 min. Thereafter, permeabilization and closure of the cells were achieved by incubating them in a solution comprising 5% BSA and 0.5% Triton‐X‐100 at room temperature for 70 min. Immunofluorescence was executed by subjecting the cells to an overnight incubation at 4°C with primary antibodies, followed by an additional hour of incubation at room temperature utilizing the corresponding secondary antibodies. Finally, the nuclei were restained with DAPI, and the images were captured using an immunofluorescence microscope. Differentiated BMSCs were also stained with the above steps.

#### 4.8.8 Transmission Electron Microscopy

Tendon–bone interface and supraspinatus muscle tissues were collected and cut into 1 mm^3^ cubes, fixed with 2.5% glutaraldehyde, dehydrated, and embedded in resin. Ultrathin sections (∼70 nm) were prepared and imaged using a transmission electron microscope. Quantitative ultrastructural analysis was performed using Origin 2019 software.

#### 4.8.9 Scanning Electron Microscopy

Demineralized samples were embedded in epoxy resin and cryosectioned to a thickness of 10 µm. The microstructure of sections was observed using a RISEMAGNA SEM system (TESCAN, Czech Republic) to evaluate surface morphology and tissue integration.

#### 4.8.10 Raman Spectroscopy

Frozen tissue sections (5 µm thick) were prepared and analyzed by Raman spectroscopy. The laser was focused on the tendon–bone interface, and spectral data were collected via linear mapping at 1 µm step intervals with 2 s exposure per point. Spectra in the range of 600–3000 cm^−^^1^ were acquired using LabSpec 6.0 software in extended scanning mode for compositional analysis.

#### 4.8.11 Biomechanical Test

For biomechanical assessment, the supraspinatus–humerus complex was harvested and securely mounted using custom clamps. A preload of 0.1 N was applied for 3 min to ensure tissue alignment. Subsequently, uniaxial tensile testing was conducted at a constant rate of 2 mm/min until tendon failure occurred. Load and displacement were recorded throughout the test, and load–displacement curves were generated to determine maximum load (N) and stiffness (N/mm) of the tendon.

#### 4.8.12 Grip Strength Test

In this study, we evaluated the grip strength of the right frontlimb in rats using a force sensor (XR501, Shanghai Xinrun Information Technology Co., Ltd., China), with the measurement method as previously described [58]. Rats were held by the nape while maintaining limb mobility, and gripped perpendicular to the direction of the force sensor, enabling them to grasp the attachment. Let the rats grasp the probe of the tensiometer, gently pull the tail to apply force to the tensiometer and record the maximum force value. Subsequently, the experimenter pulled them directly backward from the rod (parallel to the ground) at a consistent speed and force until the grip was lost. To ensure accuracy and reduce variability related to fatigue, a 2‐min rest period was maintained between each trial. The average of these three trials was then calculated for analysis. Stronger grip forces indicate stronger shoulder function in rat.

#### 4.8.13 Catwalk Test

CatWalk XT system (Noldus Information Technology, Netherlands), a fully automated and highly sensitive instrument for assessing voluntary movement and gait, was applied for gait analysis of rats. 5 rats in each group were randomly selected for the experiment on 1 and 2 M. Similar to a human clinical gait test, the system allows rodents to move autonomously within a restricted detection channel. A green LED light was emitted into the glass board, and high‐speed digital cameras 30 cm away recorded the paw prints in real‐time refracted by every contact with the glass. The intensity of the reflected light is proportional to the pressure placed on the glass. At each testing session, rats were habituated to the testing room for 30 min and the system was cleaned between each mouse tested. For each animal, three successful runs were acquired. We defined successful runs as spending longer than 2.0 s, but shorter than 10.0 s, with a maximum allowed speed variation ≤ 40%. Runs were rejected if the animal turned around. The more stable the gait of the rats, the more the contact surface and contact strength converged to normal suggesting that the rats had better motor function.

#### 4.8.14 Open Field Behavioral Test for Functional Assessment

To evaluate the spontaneous locomotor activity and anxiety‐related behavior following rotator cuff injury and repair, the Open Field Test (OFT) was conducted at pre‐defined postoperative time points (2 M). The open field apparatus consisted of a square arena (100 cm × 100 cm) with 40 cm high opaque walls. The floor was divided into a 10 × 10 grid of equal squares by white lines to define central and peripheral zones. Each rat was gently placed in the center of the arena at the beginning of the test. Behavioral activity was recorded using an automated video tracking system (e.g., SMART 3.0, Panlab) for a total of 5 min under standard lighting and ambient noise conditions. The following parameters were automatically analyzed: Total distance traveled (cm)—to reflect general locomotor activity. Number of rearing events—to assess exploratory behavior. Time spent in the central zone (%)—as an indicator of anxiety‐like behavior. Velocity (cm/s)—average movement speed. Grooming or immobility time (s)—passive behavior reflecting discomfort or pain. All rats were habituated to the test room for at least 30 min prior to testing. Between animals, the arena was cleaned with 70% ethanol to eliminate olfactory cues. All behavioral data were analyzed using automated tracking software and validated by manual scoring by a blinded observer.

#### 4.8.15 Establishment of a Mouse Rotator Cuff Tear (RCT) Model

To evaluate the regenerative potential of scaffolds in vivo, a murine model of rotator cuff tear was surgically created following previously established protocols with minor modifications. Male C57BL/6 mice (8–10 weeks old, 22–25 g) were obtained from the Animal Center of Fudan University and housed in SPF‐grade facilities. All procedures were approved by the Institutional Animal Care and Use Committee (IACUC) of Fudan University, and conducted in compliance with relevant animal welfare guidelines. Mice were anesthetized using intraperitoneal injection of 1% pentobarbital sodium (50 mg/kg). After achieving full anesthesia, the operative shoulder region was shaved and disinfected with povidone iodine and 75% ethanol. The mice were placed in lateral decubitus position to expose the surgical site. A longitudinal skin incision (∼1.0 cm) was made over the lateral shoulder. Blunt dissection was used to expose the deltoid muscle, which was gently separated to visualize the underlying supraspinatus tendon. The supraspinatus tendon was detached from its insertion on the greater tuberosity using a microsurgical scalpel and forceps, creating a complete tear. Sterilized scaffolds (Col, GO‐L/Col, GO‐M/Col, or GO‐H/Col) were trimmed to appropriate size (∼2 mm × 2 mm) and placed at the tendon‐bone interface. The tendon was then repositioned over the scaffold to simulate tendon‐to‐bone contact. No sutures were applied to fix the scaffolds due to their conformability and the limited space. In the blank control group, the tear was left unrepaired. Muscle and skin were closed sequentially using 6‐0 absorbable sutures. Mice were placed in a warm environment for recovery and returned to individual cages postoperatively. No immobilization devices were used. Analgesics (e.g., meloxicam, 1 mg/kg) were administered for three consecutive days post‐surgery to minimize discomfort. Mice were monitored daily for signs of infection, wound healing, and general health status. At 2, 4, or 8 weeks post‐operation (depending on study design), mice were euthanized for histological and biomechanical evaluations of the tendon‐to‐bone healing interface.

#### 4.8.16 mRNA Sequencing and Bioinformatics Analysis

At the culmination of the 3 days following the application of treatment, samples of mouse TBI tissue were meticulously harvested for mRNA sequencing. Cell samples were collected after different treatments. The process of isolating total RNA from the samples was carried out employing the esteemed RNeasy Mini Kit (Qiagen, Hilden, Germany). Subsequently, the construction of paired‐end libraries was accomplished utilizing the TruSeq RNA Sample Preparation Kit (Illumina, USA), in strict accordance with the TruSeq RNA Sample Preparation protocol. The esteemed Shanghai Biotechnology Corporation was entrusted with the responsibility of library construction and sequencing. In order to accurately map the clean reads to the Rnor 6.0 reference genome, with an allowance for two mismatches, the renowned Hisat2 software (version 2.0) was employed. Following genome mapping, the generation and annotation of Fragments per kilobase of exon per million (FPKM) values were carried out using the esteemed Stringtie software (version 1.3.0). To establish statistical significance, the *p*‐value threshold was determined based on the false discovery rate (FDR). Upon analysis, mRNAs displaying a fold change of ≥2 and an FDR ≤ 0.05 were deemed to be differentially expressed. Differential expression analysis was performed using the DESeq2 package in R. To further elucidate the underlying biological pathways, a KEGG pathway analysis was meticulously performed, utilizing the esteemed KEGG database (http://www.genome.ad.jp/kegg) and Gene ontology enrichment analysis in the R environment. GSEA was conducted using R BiocManager.

#### 4.8.17 Inflammation Antibody Assay

For the assessment of the inflammatory milieu following RCT repair in murine models, we engaged a robust Mouse Inflammation Antibody Array (Catalog no. ab133999, Abcam) capable of simultaneously detecting 40 distinct inflammatory markers. Our methodology adhered stringently to the supplier's protocol to ensure the integrity and reproducibility of the results. In summary, the assay commenced with a 1‐h blocking phase of the antibody array membranes using a 2 mL proprietary blocking buffer. This step was succeeded by an overnight incubation at 4°C with the sample/antibody cocktail. Subsequent to the removal of the incubation mixture, a trio of washes were conducted at room temperature to eliminate non‐specific binding. The membranes were then exposed to streptavidin‐conjugated horseradish peroxidase, diluted 1:1000, for 1 h at ambient temperature. Post‐wash, we advanced to the chemiluminescent detection phase, ensuring a thorough final rinse to minimize background interference. The resultant chemiluminescent signals were captured via imaging, culminating in the visualization and quantification of the array's inflammatory targets.

#### 4.8.18 Mass Cytometry (CyTOF)

Mass cytometry analysis also recognized as CyTOF, was executed according to an optimized procedure derived from established protocols [59]. Cells, harvested from mouse TBI tissue or blood specimens, were subjected to a 15‐min barcoding stage in Maxpar Barcode Perm Buffer (Catalog number: 201067, Fluidigm Corporation, USA), during which they were tagged with exclusive combinations of isotopically pure palladium ions, each chelated to isothiocyanobenzyl‐EDTA. Post‐labeling, the cellular samples were treated with an Fc Receptor Blocking Solution (Catalog number: abs9477, Absin Bioscience Inc., China) to mitigate unspecific antibody adherence. This step preceded a 30‐min antibody cocktail incubation designed to selectively bind to cellular epitopes. Following the incubation, cells underwent a permeabilization process with cold methanol and were stained with an array of intracellular markers for an hour. The stained cells were then introduced to overnight incubation with an iridium‐containing intercalating agent (191Ir/193Ir), supplied by DVS Sciences, in a formaldehyde solution to fix the cells. Before the final analysis, calibration beads were added to ensure accuracy in the mass cytometry assessment performed on a Helios system (Catalog number: Helios Mass Cytometer, Fluidigm Corporation, USA). Approximately half a million cells from each sample were acquired at a throughput of 400 to 500 events per second. For data interrogation, we utilized the Cytobank platform (version 8.1, Beckman Coulter), applying the opt‐SNE algorithm for high‐dimensional data reduction and spatial visualization. A diverse set of 24 antibodies was employed for the comprehensive analysis as delineated in Table S3.

### 4.9 Biocompatibility of GO/Col Scaffold In Vivo

#### 4.9.1 Acute Toxicity Test

To ascertain the biosafety of GO/Col scaffold, an intricate experimental procedure was carried out [60, 61, 62]. At the designated time points of 14 and 28 days postoperatively, the TBI tissue and skeletal muscle tissue near the wound were meticulously excised, accompanied by the removal of portions of the heart, liver, spleen, lungs, and kidneys. These excised tissues were carefully cleansed and subsequently immersed in a 4% paraformaldehyde solution for a minimum of 24 h to ensure optimal fixation. Following fixation, the samples were subjected to a meticulous dehydration process and thereafter embedded in paraffin to generate precise 5 µm sections. These sections were then subjected to a controlled dewaxing and rehydration process, employing a careful gradient technique utilizing xylene/alcohol and PBS solutions. To comprehensively evaluate the biosafety of the material, a series of staining techniques including HE staining, Masson's trichrome staining, and immunofluorescence staining were meticulously applied to the sections.

#### 4.9.2 Subcutaneous Degradation Test

To evaluate the in vivo biocompatibility of the scaffold, GO/Col composites (2 mm × 2 mm × 1 mm) were bilaterally implanted subcutaneously into the dorsal region of 8‐week‐old Sprague‐Dawley rats. Postoperatively, each rat received intramuscular penicillin injections (40 000 IU/100 g body weight) in the thigh for three consecutive days to prevent infection. Daily assessments were conducted to monitor general health, including activity, gait, respiration, and neurological responses, as well as signs of local wound complications such as erythema, ulceration, or abnormal discharge. Animals were euthanized at 1, 2, and 4 weeks post‐implantation for histological evaluation of the surrounding tissue.

#### 4.9.3 Coagulation Assay

A coagulation assay was performed using freshly collected anticoagulated rat whole blood. The blood was mixed with 0.1 M calcium chloride to initiate coagulation. Subsequently, 50 µL of the mixture was added to each well of a 96‐well plate. Wells were thoroughly rinsed with 0.9% saline solution until the wash appeared clear, ensuring removal of uncoagulated components. Test materials were placed at the bottom of each well to allow direct contact with the blood; wells containing blood alone served as blank controls. All experiments were conducted in triplicate to ensure data reliability.

#### 4.9.4 CyTOF Analysis of Blood

Whole blood from mice implanted with different materials (see modeling details) was pooled from three animals per group to generate one biological sample. After red blood cell lysis, samples were preserved at 4°C. Cells were first barcoded using Maxpar Barcode Perm Buffer containing isotopically pure palladium ions chelated with benzyl‐isothiocyanate‐EDTA for 15 min. Fc receptor blocking solution was applied to minimize nonspecific antibody binding. Subsequently, cells were incubated with a surface antibody cocktail (listed in Table S3) for 30 min, permeabilized with cold methanol, and stained with intracellular markers for 1 h. The stained cells were fixed overnight using 191Ir/193Ir‐labeled DNA intercalators in formaldehyde. Calibration beads were added before acquisition to ensure data accuracy. Approximately 500 000 events per sample were collected using a Helios mass cytometer at a rate of 400–500 events per second. Data were uploaded to Cytobank and analyzed using the SPADE algorithm to identify immune cell subpopulations and calculate their relative proportions based on marker expression.

### 4.10 Statistical Analysis

All experiments were independently repeated at least three times unless otherwise stated. Data are presented as mean ± standard deviation (SD). Statistical analyses were performed using GraphPad Prism 9.0 (GraphPad Software, CA, USA). Before inferential testing, data were assessed for normality using the Shapiro–Wilk test and for homogeneity of variance using the F test (for two‐group comparisons) or Brown–Forsythe test (for multiple‐group comparisons), where applicable. No data transformation was applied unless otherwise specified. Outliers were not excluded unless predefined technical failure criteria were met. For comparisons between two groups, an unpaired two‐tailed Student's t‐test was used for normally distributed data, whereas the Mann–Whitney U test was used for non‐normally distributed data. For comparisons among three or more groups, one‐way ANOVA or two‐way ANOVA was applied as appropriate, followed by Bonferroni's post hoc multiple‐comparisons test when applicable. The sample size (n) for each statistical analysis is indicated in the corresponding figure legends. A two‐sided *p* < 0.05 was considered statistically significant.

## Author Contributions

R.W., Y.D., C.C., Y.H., X.F., and Z.L. conceived and designed the study. R.W., Y.D., Y.H., X.F., W.L., Z.F., Z.H., K.F., X.L., D.W., and B.H. conducted scaffold preparation, physicochemical characterization, and related analyses. R.W., C.C., Y.C., Z.Z., J.M., Z.L., and Z.L. performed animal experiments and histological evaluations. R.W., Y.D., Y.H., X.F., Z.H., K.F., and X.L. executed multi‐omics analyses and bioinformatics. R.W., Y.D., C.C., Z.L., J.M., and B.H. were involved in biomechanical tests and functional assessments. R.W., C.C., Y.C., Z.Z., J.M., and W.L. contributed significantly to data collection, statistical analysis, and data interpretation. R.W., Y.D., C.C., Y.H., X.F., and Z.L. drafted and edited the manuscript. J.H.H., S.C., G.C., N.B., J.Q., and Z.L. provided critical resources, supervision, manuscript revision, and funding support. All authors reviewed and approved the final manuscript.

## Conflicts of Interest

The authors declare no conflict of interest.

## Supporting information




**Supporting File**: advs75683‐sup‐0001‐SuppMat.pdf.

## Data Availability

All data are available in the main text or the supplementary materials. The materials generated are available from the lead contact upon reasonable request.
