# Supplementary material for: Multi‐Omics Profiling Reveals Immunomodulatory and Pro‐Regenerative Effects of a Graphene Oxide–Collagen Scaffold in Massive Rotator Cuff Tears
Source: Adv Sci (Weinh). 2026 May 21;13(44):e23821. doi: 10.1002/advs.202523821 (PMC13335791; doi:10.1002/advs.202523821)
Supplement: Supplementary file 1 [file ADVS-13-e23821-s001.pdf]

Supplementary Materials for

Multi-omics Profiling Reveals Immunomodulatory and Pro-regenerative Effects of a Graphene Oxide–Collagen Scaffold in Massive Rotator Cuff Tears

Authors: *Renwen Wan[1,2,3#], Yechuan Deng[4#], Zixin Hu[5,6#*], Yanwei He[1#], Xinting Feng[1#], Jun Ma[1,2], Yisheng Chen[7,8,9], Zhijie Zhao[10], Jie Mei[11], Zhiheng Lin[12], Wei Luo[1], Zhengyuan Fang[13], Zhufeng Hu[14], Kunlun Feng[15], Xinrong Li[16], Dan Wu[14], Bowen Han[15], James Hoipo Hui[16], Xuanyong Liu[4], Chen Chen[17], Gang Chen[1,2*], Shiyi Chen[1*], Nirong Bao[18*], Jiajun Qiu[4*], and Zhiwen Luo[1,2,3,16, 19*]*

Corresponding author: adcyy@aliyun.com, huzixin@fudan.edu.cn, cshiyi@163.com, bao.nirong@nju.edu.cn, qiujiajun@mail.sic.ac.cn, zhiwen.luo_fudan@hotmail.com

The PDF file includes:

Materials and Methods
Figs. S1 to S12
Tables S1 to S4
References

Other Supplementary Materials for this manuscript include the following:

Sup Figures:

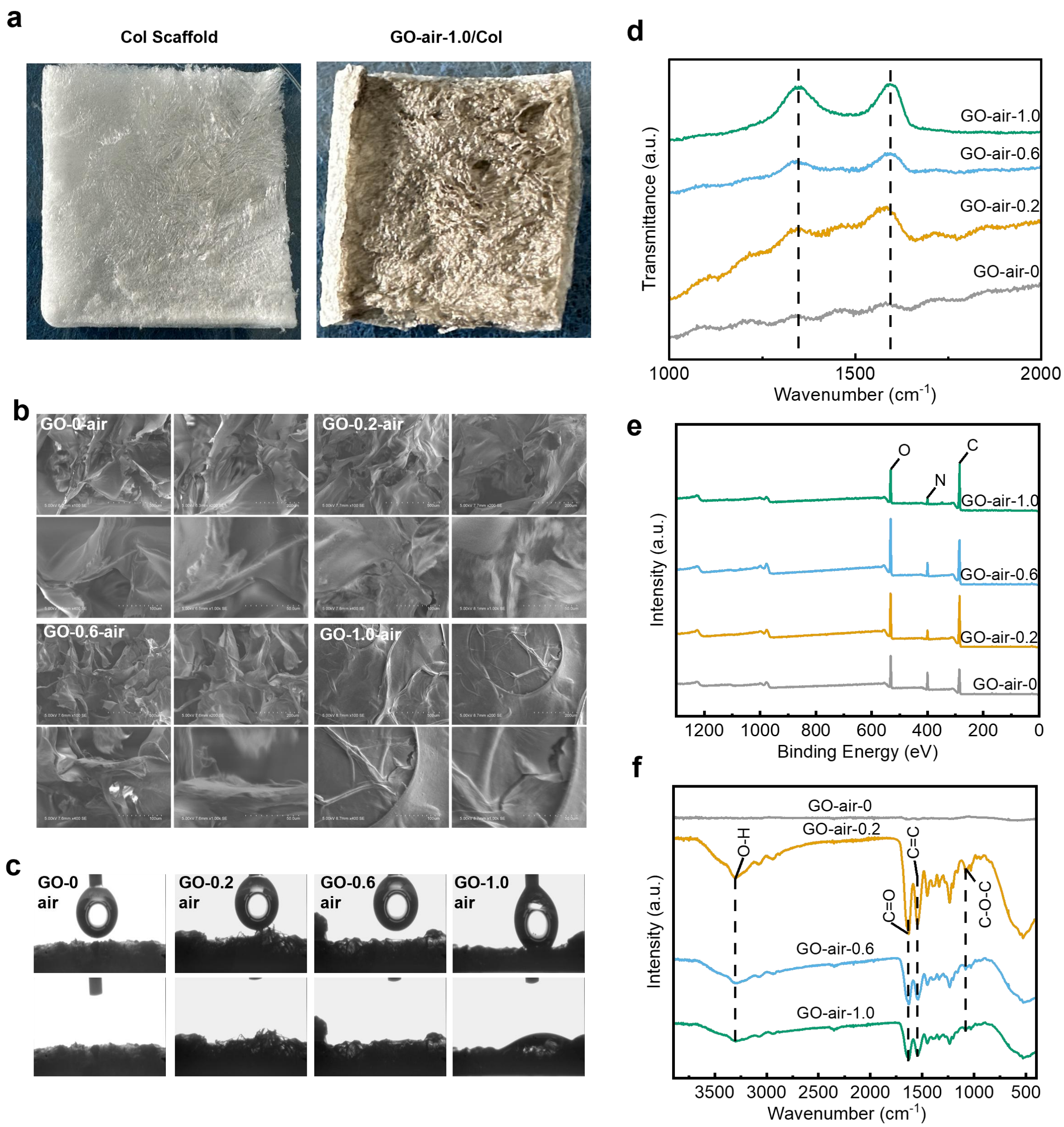

Supplementary Figure 1. Characterization of the air-dried GO/Col-air scaffold.
(a) Gross morphology of the scaffold.
(b) Scanning electron microscopy (SEM) images at scale bars of 500 µm, 200 µm, 100 µm, and 50 µm.
(c) Sample surfaces before (up) and after (down) water contact angle measurement using sessile drop method.
(d) Raman spectroscopy analysis.
(e) X-ray photoelectron spectroscopy (XPS) analysis.
(f) Fourier-transform infrared spectroscopy (FTIR) analysis.

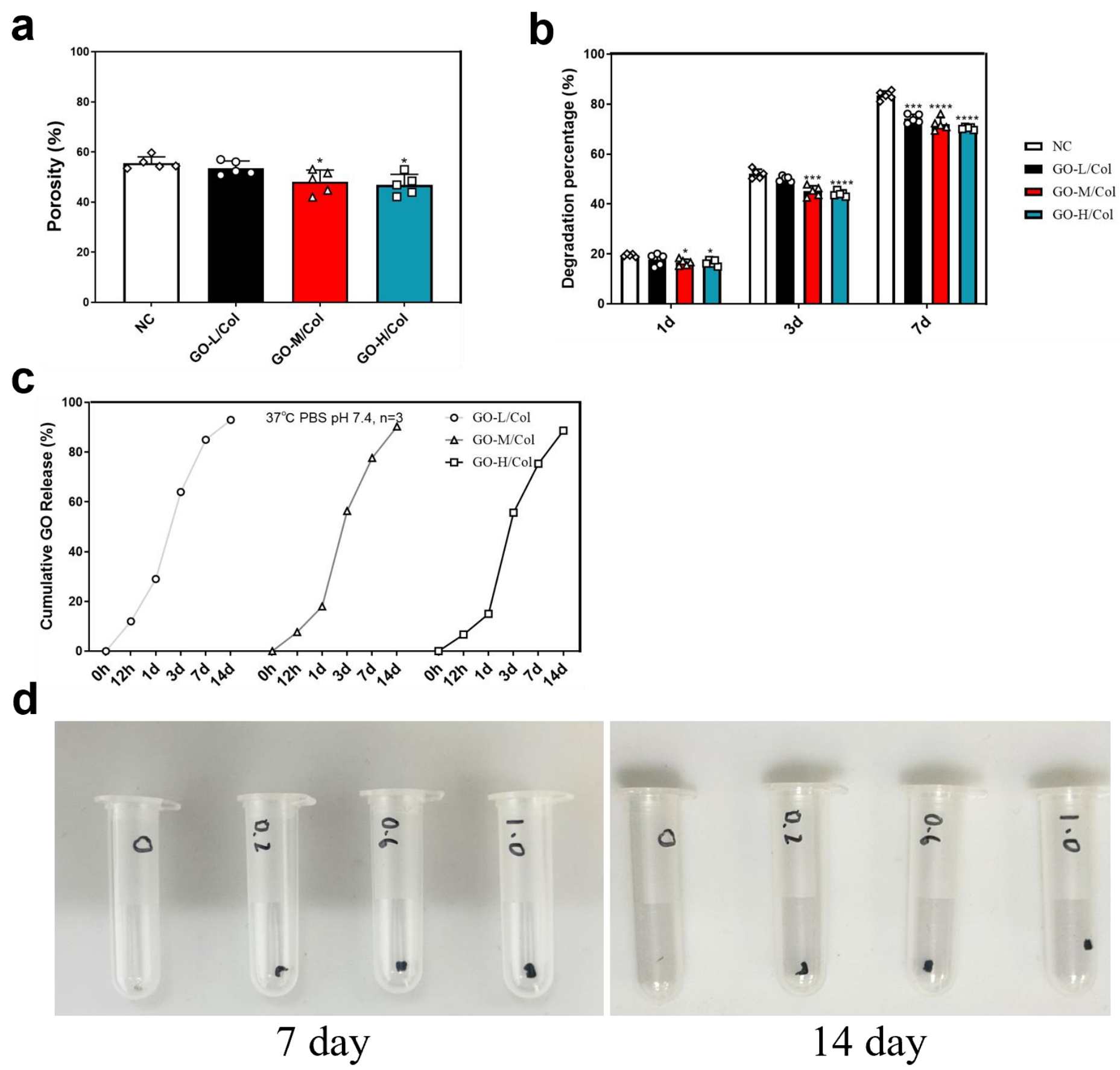

Supplementary Figure 2. Biodegradability of the GO/Col scaffold.
(a) Quantitative analysis of porosity for scaffolds with different GO concentrations (n = 5, P < 0.05).
(b) Degradation profiles of GO/Col scaffolds incubated in PBS at 37 °C for 1, 3, and 7 days.
(c) GO release from the scaffold under PBS at 37 °C at time points: 0 h, 12 h, 1 d, 3 d, 7 d, and 14 d.
(d) Photographic records of scaffold degradation at days 7 and 14 (n = 5; P < 0.05; **P < 0.001; ***P < 0.0001). Col=Col Scaffold; Col-0.1=Col-L; Col-0.6=Col-M; Col-1.0=Col-H.

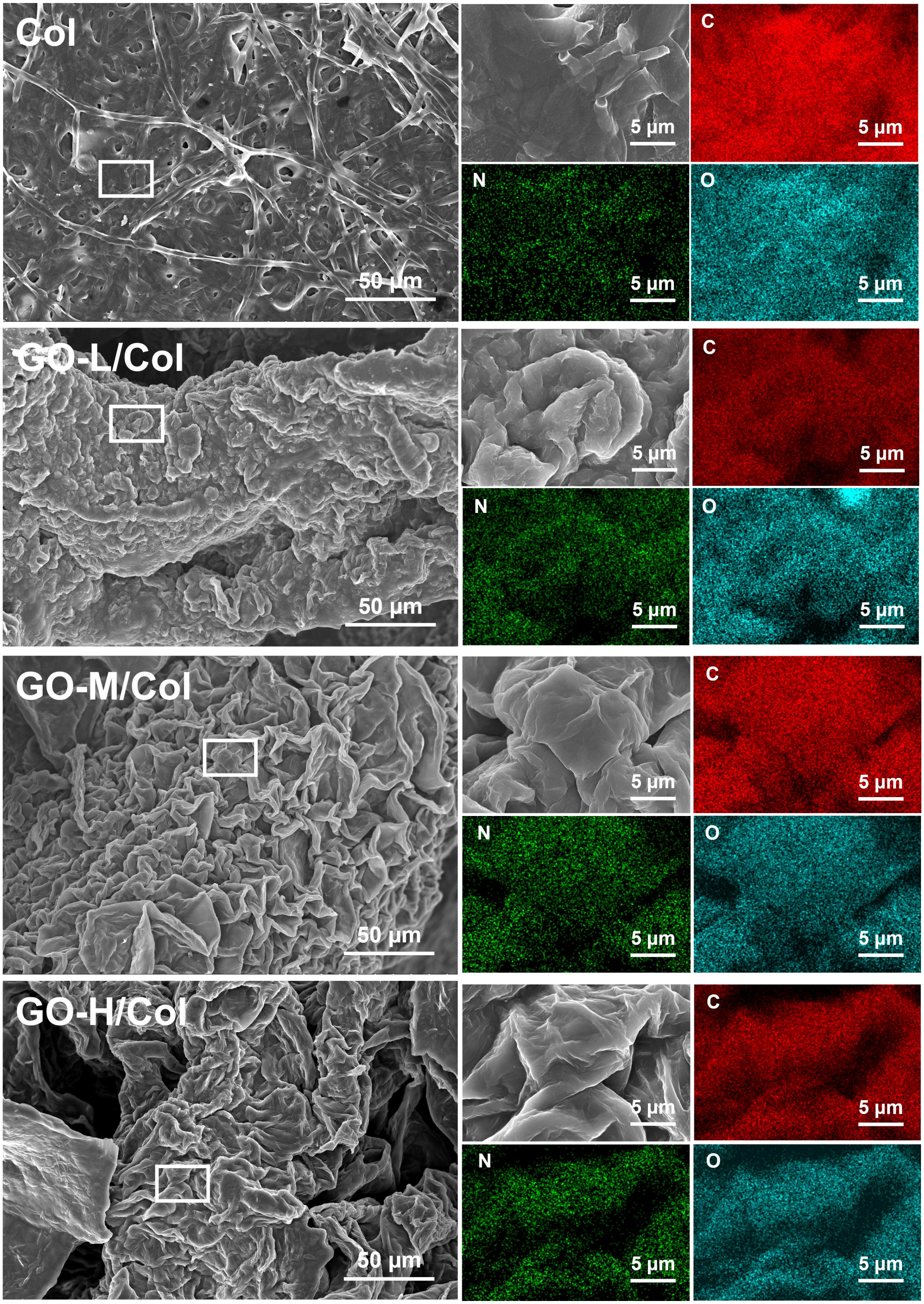
Col
50 μm
5 μm
C
N
O
GO-L/Col
GO-M/Col
GO-H/Col

Supplementary Figure 3. The GO/Col scaffold retains its 3D architecture during degradation.
SEM and elemental composition images of scaffolds after 14 days of degradation. Scale bars: 50 μm and 5 μm.

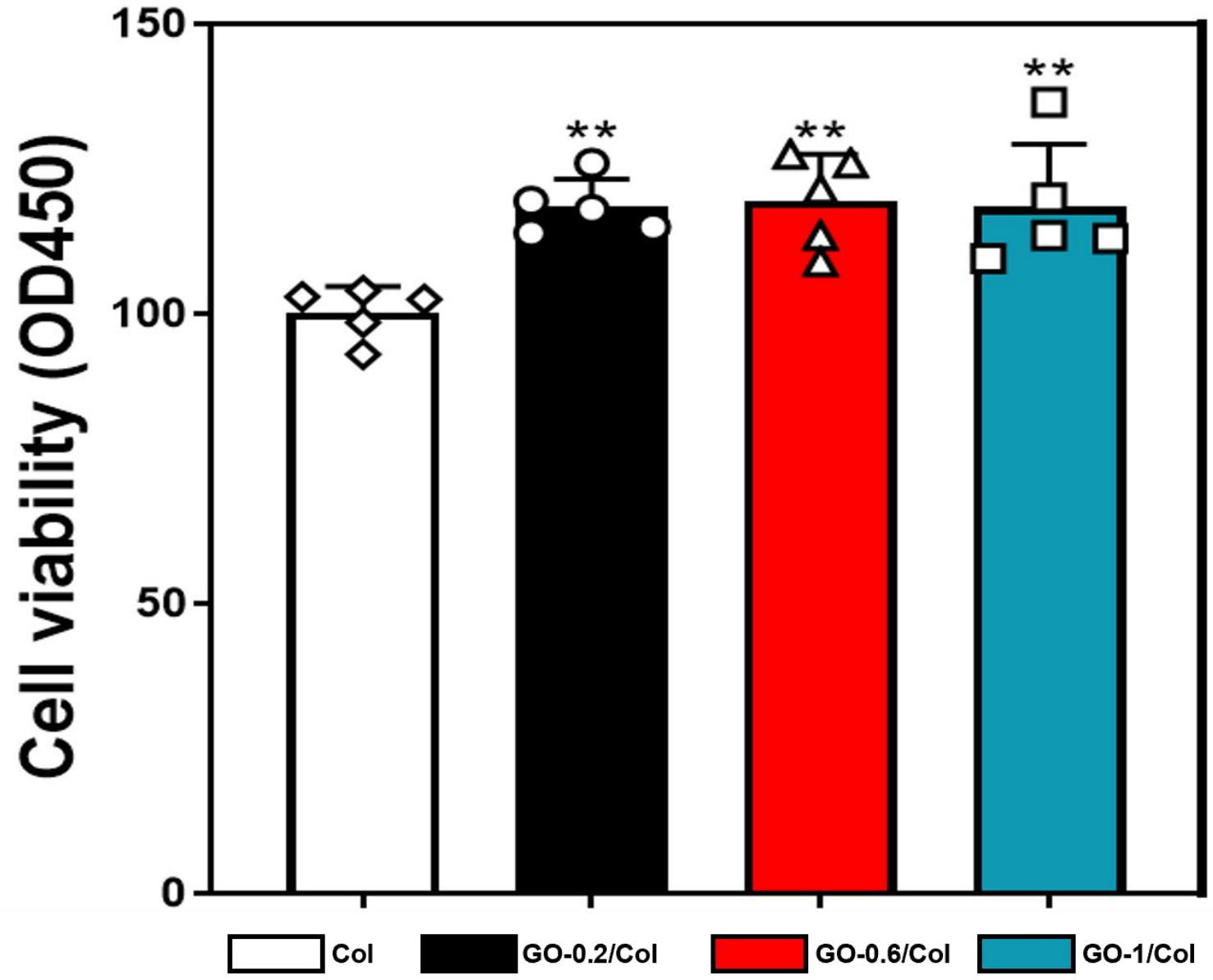

Supplementary Figure 4. The GO/Col scaffold promotes BMSC viability.
(a–b) CCK-8 assay results at 24 hours (n = 5; P < 0.05; *P < 0.01; ***P < 0.0001). Col=Col Scaffold; Col-0.1=Col-L; Col-0.6=Col-M; Col-1.0=Col-H.

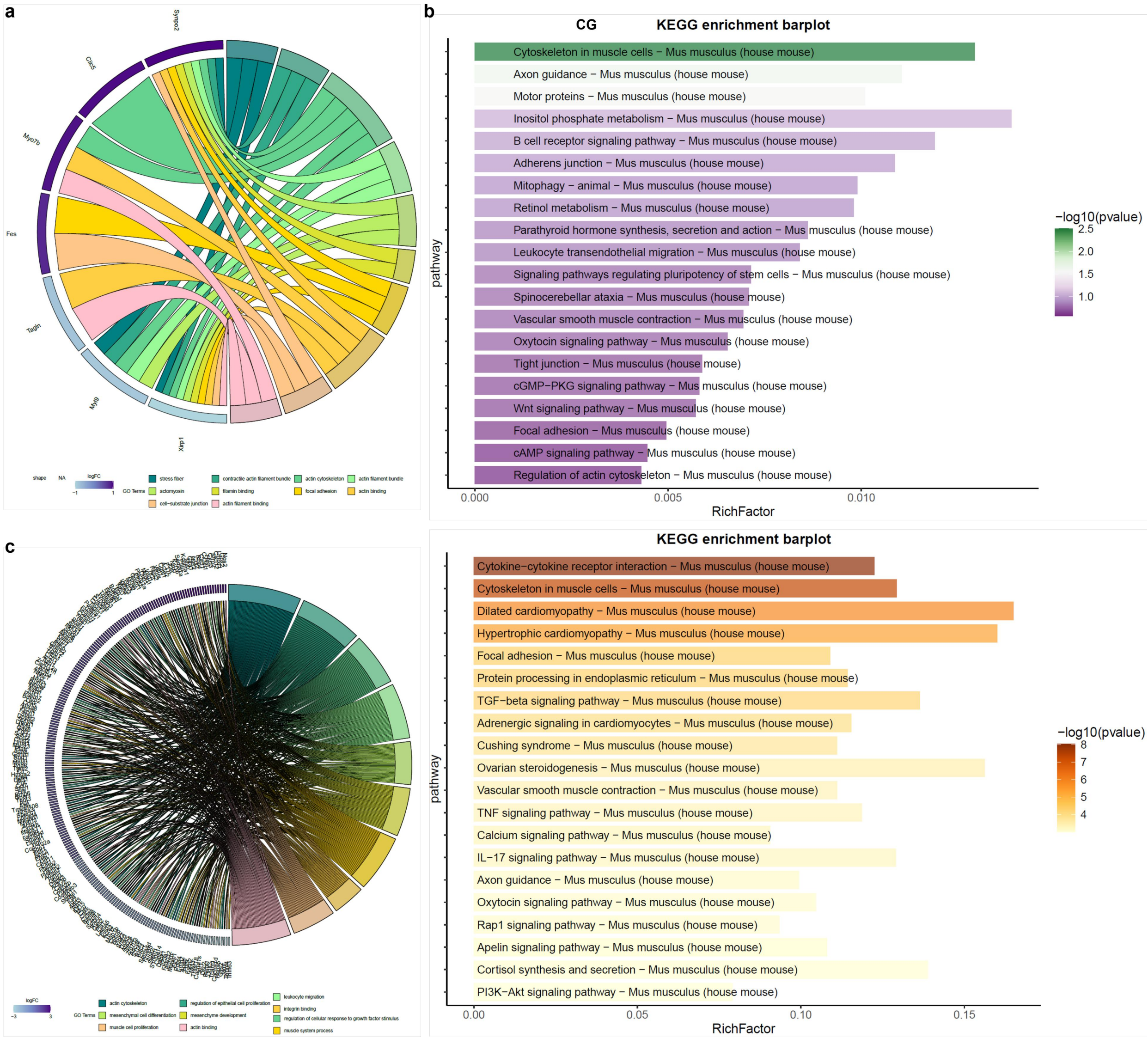

Supplementary Figure 5. Additional bioinformatic analysis related to Figure 3.
(a–c) Gene correlation analysis and KEGG pathway enrichment analysis.

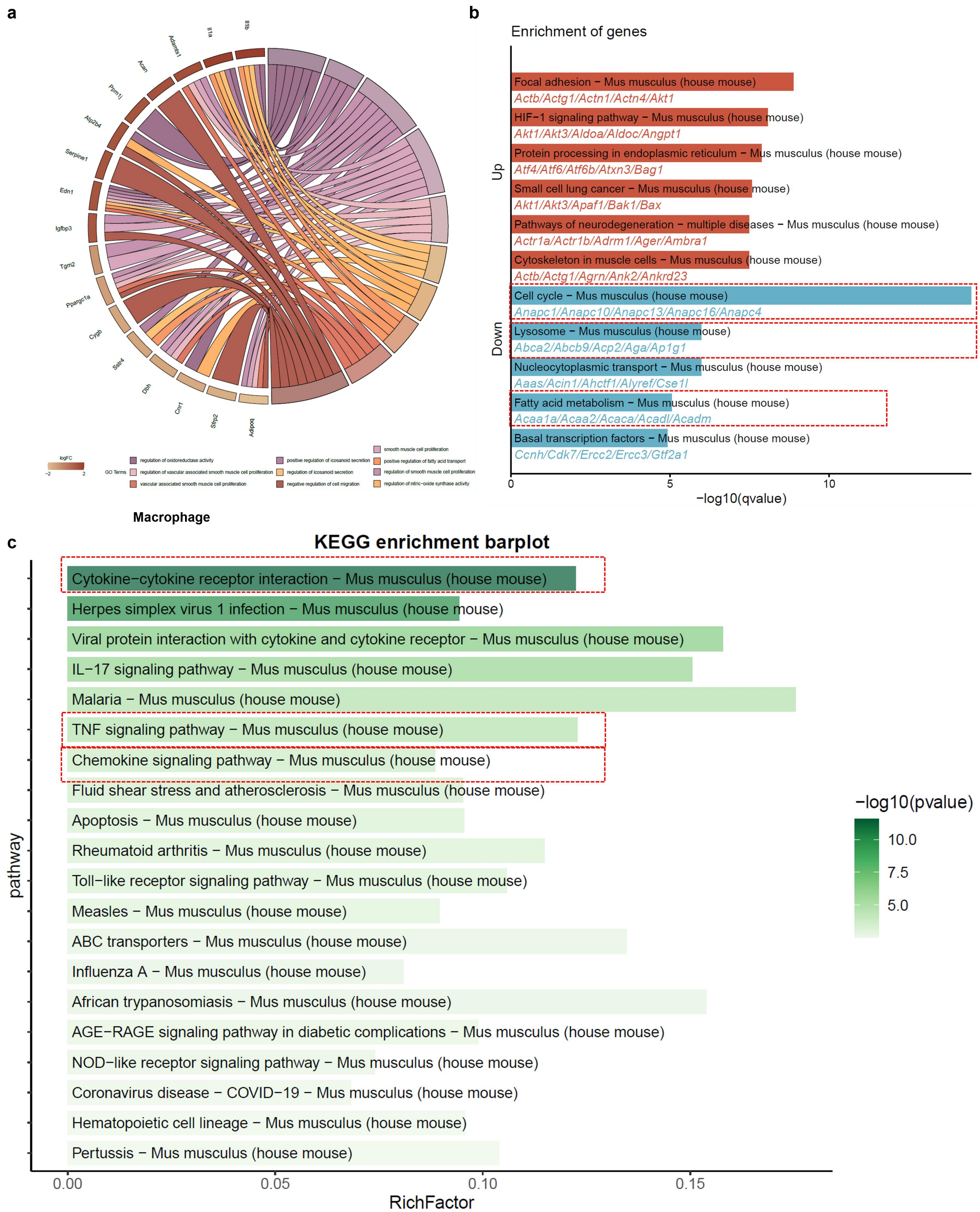

Supplementary Figure 6. Additional bioinformatic analysis related to Figure 4. (a–c) Gene correlation analysis and KEGG pathway enrichment analysis.

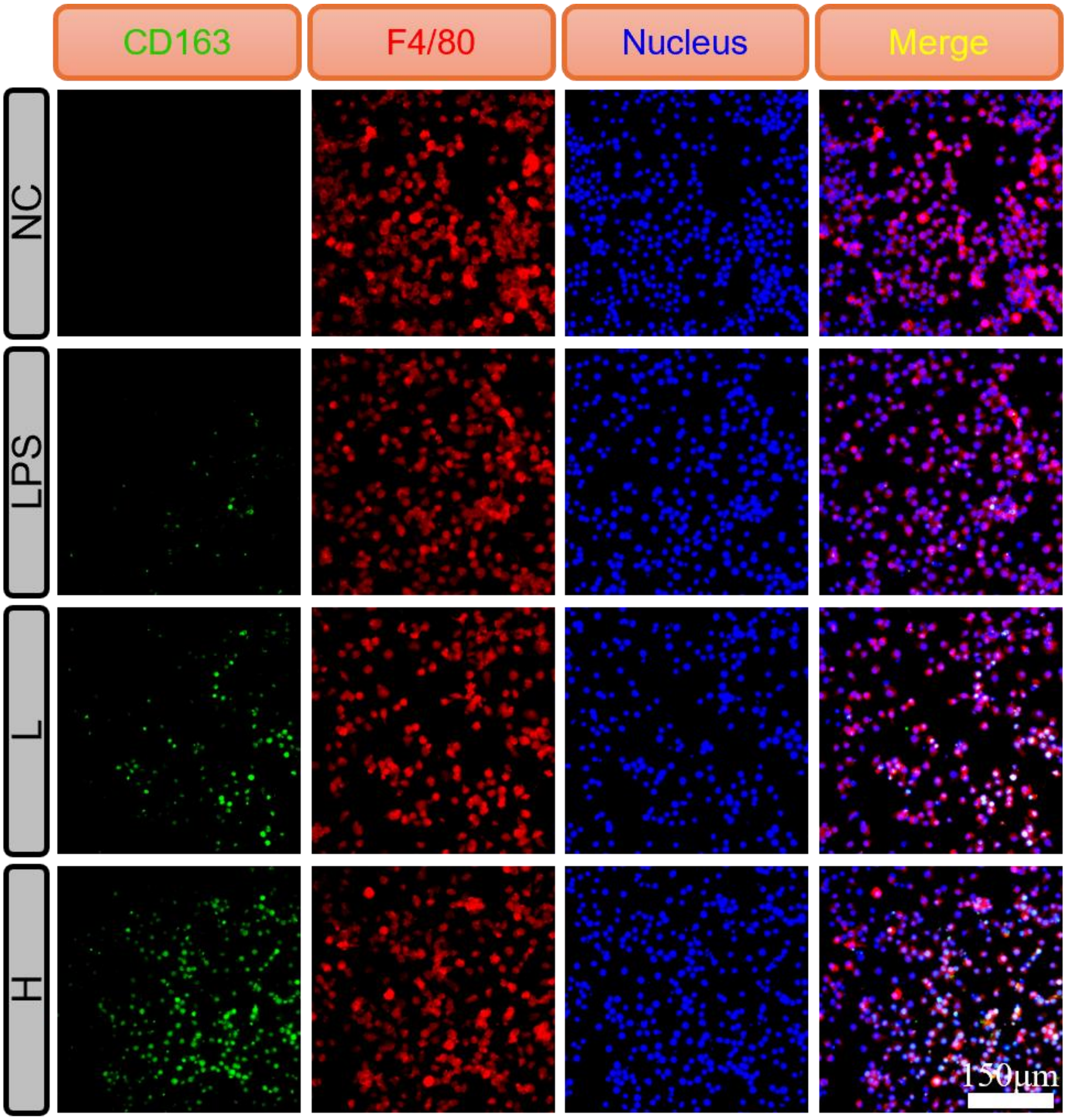

Supplementary Figure 7. Additional analysis of macrophage polarization regulated by the GO/Col scaffold.

Immunofluorescence staining for RAW264.7 macrophages cultured on scaffolds with different GO concentrations for 24 hours. CD163+ (green) indicates M2 polarization; F4/80 (red) marks macrophages; DAPI (blue) stains nuclei. Col=Col Scaffold; Col-0.1=Col-L; Col-0.6=Col-M; Col-1.0=Col-H.

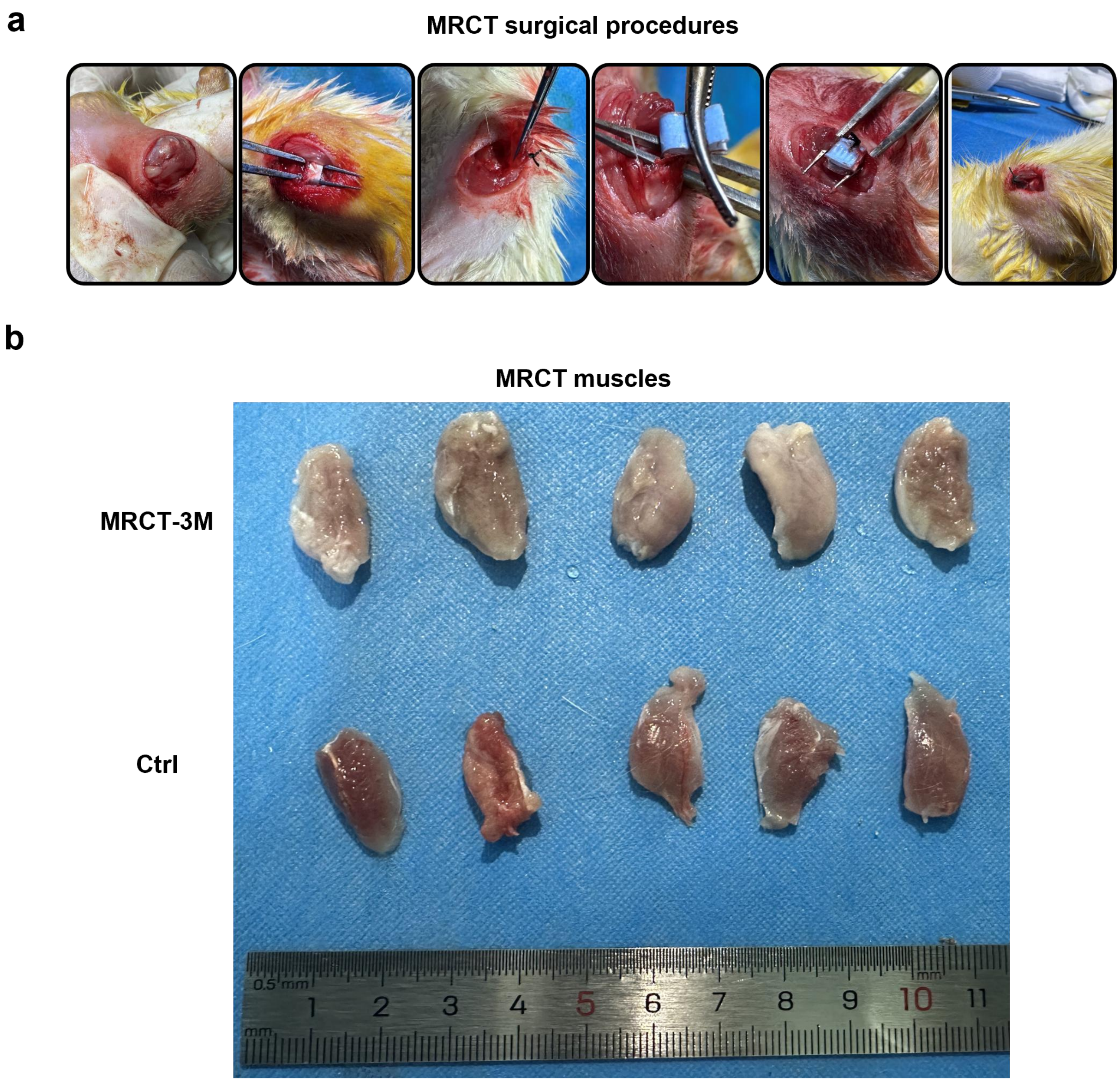

Supplementary Figure 8. Construction and validation of the chronic MRCT rat model.
(a) Photographs illustrating the surgical procedure for establishing the chronic massive rotator cuff tear (MRCT) model.
(b) Gross comparison of supraspinatus muscle morphology at 3 months post-MRCT.

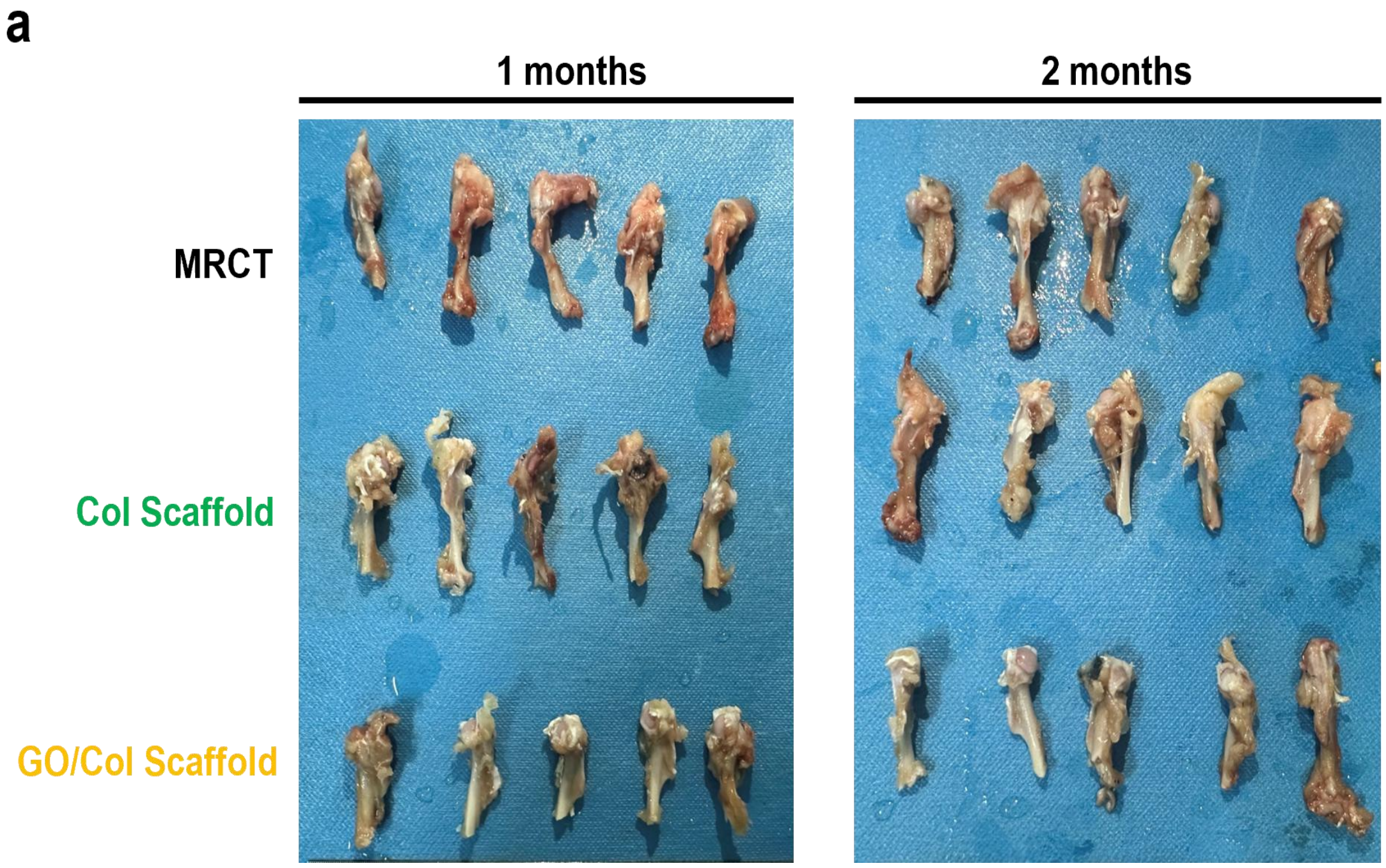

Supplementary Figure 9. Gross histological images of shoulder joints related to Figure 6.
(a) Representative histological images of shoulder joints at 1 and 2 months post-MRCT repair.

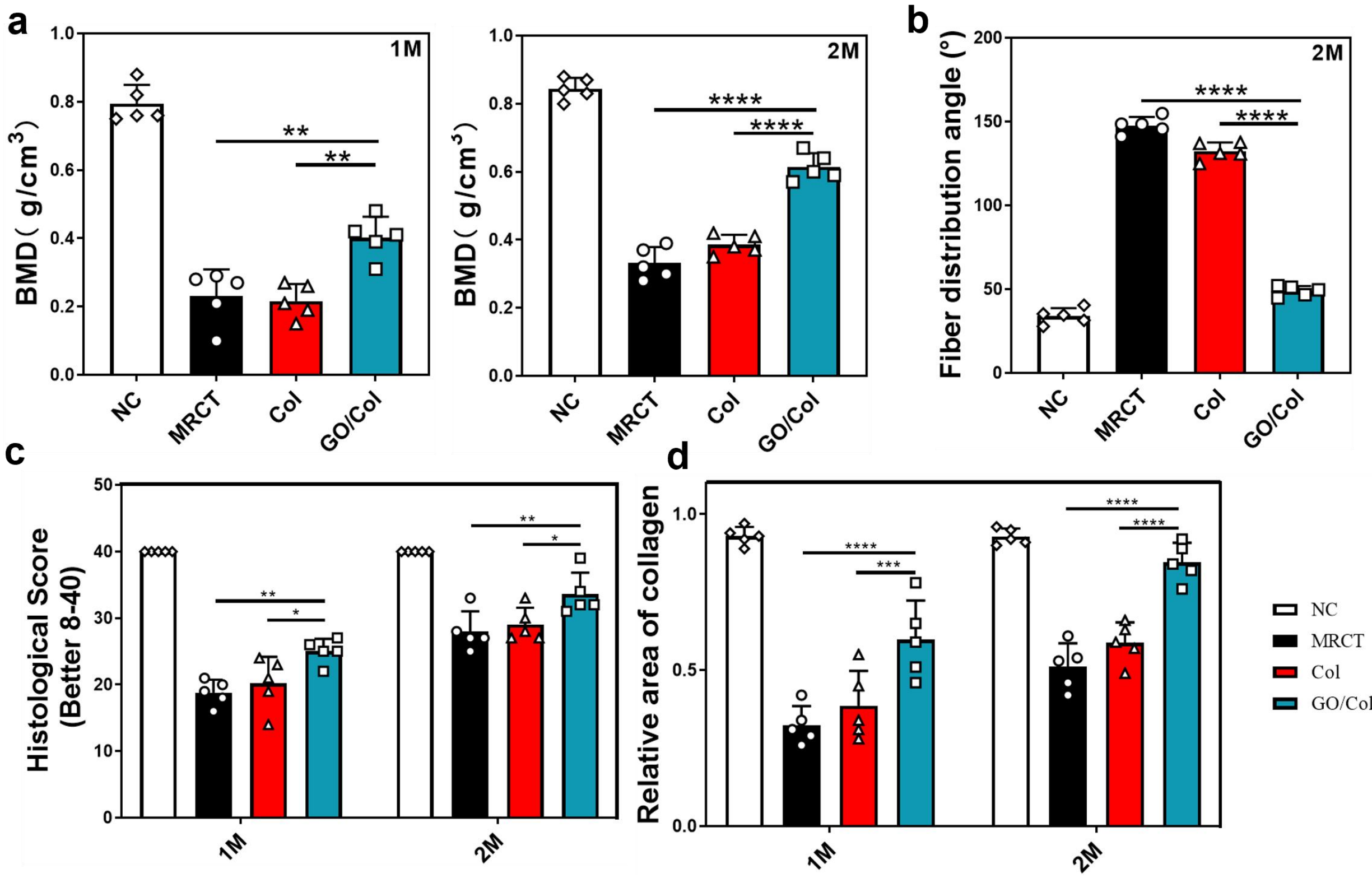

Supplementary Figure 10. Histological analysis related to Figure 6.

(a–b) Quantification of bone mineral density (BMD) at the tendon-bone interface and tendon alignment area.

(c) Histological scoring of H&E-stained sections.

(d) Safranin O–Fast Green staining-based cartilage collagen score. (NC: sham; MRCT: repair only; Col: repair + Col scaffold; GO/Col: repair + GO/Col scaffold; n = 5; P < 0.05; *P < 0.01; ***P < 0.0001).

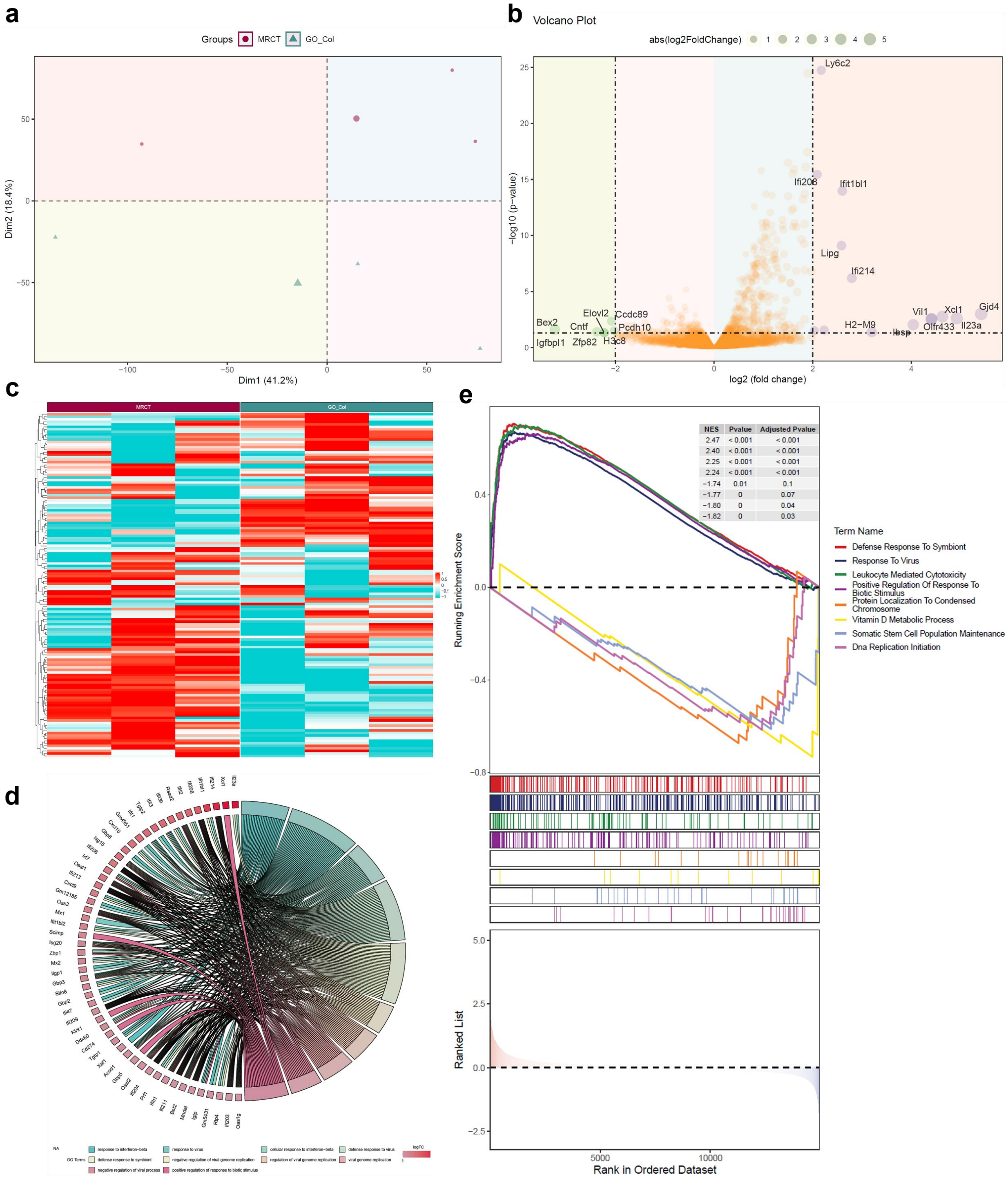

Supplementary Figure 11. Transcriptomic sequencing analysis supplementing Figure 8.
(a) Principal component analysis (PCA).
(b) Volcano plot of differentially expressed genes.
(c) Gene expression heatmap.
(d) Gene-to-pathway correlation analysis.
(e) Gene Set Enrichment Analysis (GSEA).

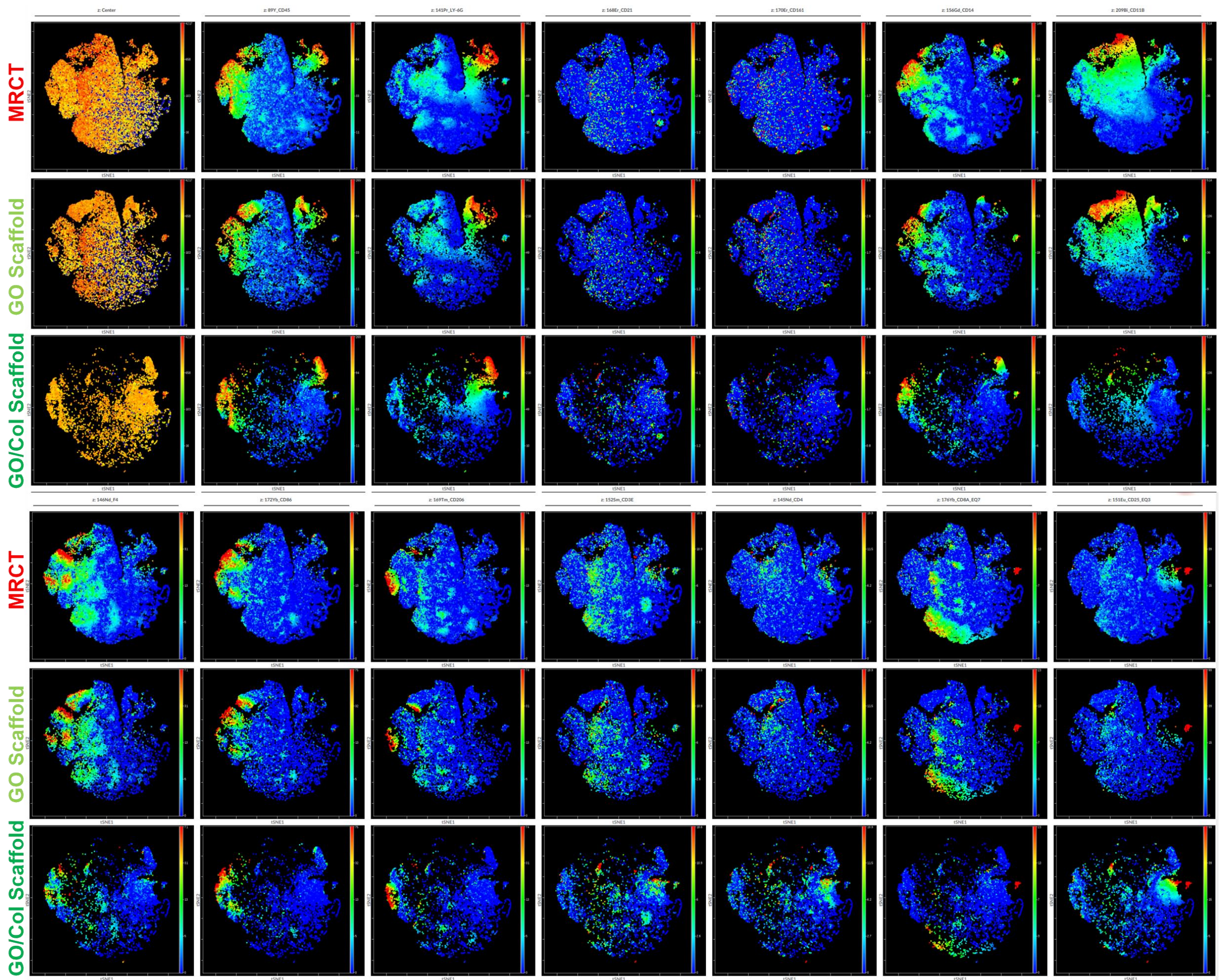

Supplementary Figure 12. CyTOF analysis supplementing Figure 8.
Dimensional reduction and expression patterns of various protein markers across cell clusters in different treatment groups.

Sup Tables:

Supplemental Table 1. Primary antibodies used in the experiment

Antibody	Source	Catalog No.	Type	Dilution	M.W. (kD)
iNOS	Abcam	ab178945	Rabbit mAb	1:200(IF)	130
Arg1	Affinity	DF6657	Rabbit mAb	1:2000(IF)	35
CD86	Abcam	ab220188	Rabbit mAb	1:100(IF)	38
CD86 PE	eBioscience	12-0862-81	Rat mAb	0.125 μg/test	(Flow Cyt)
CD163	Abcam	ab64693	Rabbit PAb	1:200(IF)	150
CD163APC	eBioscience	17-1631-80	Rat mAb	0.25μg/test	(Flow Cyt)
MYHC	Abcam	ab51263	Mouse mAb	1:500(IF)	227
MyoD1	Proteintch	18943-1-AP	Rabbit PAb	1:200(IF)	60
PAX7	Santa Cruz	SC-81648	Mouse mAb	1:20(IF)	55
Lamin B1	Abcam	ab16048	Rabbit PAb	1:100(IF)	66
Collagen 1	Affinity	AF7001	Rabbit mAb	1:200(IF)	140
a-SMA	Abcam	ab7817	Mouse mAb	1:200(IF)	42
F4/80 APC	eBioscience	47-4801-80	Rat mAb	0.125μg/test	(Flow Cyt)
F4/80	Abcam	ab6640	Rat mAb	1:200(IF)	160
MHC I (BA-D5)	DSHB	AB2235587	Mouse mAb	1:200(IF)	222.9
MHC IIA(SC-71)	DSHB	AB2147165	Mouse mAb	1:200(IF)	223
MHC IIB(BF-F3)	DSHB	AB2266724	Mouse mAb	1:200(IF)	223
MHC IIX (6H1)	DSHB	AB2314830	Rabbit mAb	1:200(IF)	200
RUNX2	Abcam	ab192256	Rabbit mAb	1:200(IF)	
COL-1	Abcam	ab270993	Rabbit mAb	1:200(IF)	
COL-2	Abcam	ab34712	Rabbit PAb	1:200(IF)	
COL-3	Abcam	ab184993	Rabbit mAb	1:200(IF)	
PPARγ	Abcam	ab272718	Rabbit mAb	1:200(IF)	
α-SMA	Abcam	ab40854	Rabbit mAb	1:200(IF)	
SOX9	Abcam	ab185966	Rabbit mAb	1:200(IF)	
PCNA	Abcam	ab29	Mouse mAb	1:200(IF)	
CD44	Abcam	ab238464	Mouse mAb	1:200(IF)	
Aggrecan	Abcam	ab3773	Rabbit mAb	1:200(IF)	

Supplemental Table 2. Primers for PCR used in the experiment

Name	Forward	Reverse	大小
H-GAPDH	GGAAGCTTGTCATCAATGGAAATC	TGATGACCCTTTTGGCTCCC	168 bp
H-NOS2	TTCACCATAAGGCCAAAGGGA	CTTTGTTACCGCTTCCACCCT	215 bp
H-CXCL6	ACTATGAGCCTCCCGTCCAG	CTGTCAGCACAGCAGAGACA	139 bp
H-MYOD1	CGACGGCATGATGGACTACAG	AGGCAGTCTAGGCTCGACACC	135 bp
H-PPARGC1A	CTGACCACAAACGATGACCCTC	ATCTTGGTTGGCTTTATGAGGAG	282 bp
H-AQP7	TCCAGGAAATACTGCAGAGGAAGA	AAGCCAAAACCCAAGTTGACAC	154 bp
H-ADIPOQ	TGTTGCTGGGAGCTGTTCTACTG	CGATGTCTCCCTTAGGACCAAT	237 bp
H-SFRP2	ACATGCTTGAGTGCGACCGT	GCAGGCTTCACATACCTTTGGA	113 bp
H-OLFML2B	CCTCCCACCACAGTCAGAACAG	TCCTTGCACCTTCCTATGACATTC	131 bp
H-CIDEC	ATCAGAACAGGGGACAAGGCA	AGTGCAGATCATAGGAAAGGGAGTA	173 bp
H-ANO1	ACTGAAGATGCCGACGAAGAAG	TTCAAGATCTCATAGACAATCGTGC	246 bp
H-GBX2	GTAACTTCGACAAGGCGGAGG	GTCTTCCACCTTTGACTCGTCTTTC	201 bp
H-GDF6	TTCCAGTCTTCCAAGTCGGC	GAGGAGTGTGCGAGAGATCG	76 bp
H-ERG	CAGCCAGGGTCACCATCAAA	CCACCATCTTCCCGCCTTT	102 bp
H-FES	CATTCCTTTGCTCATCGACCAC	GTCCAATCTGCTCACCCAACA	140 bp
H-Tent5c	GGACCTAATCTTCCATGTGGCTCT	TGAACTCAAACTGACGCCGAAT	266 bp
M-Apoe	GTGCTGTTGGTCACATTGCTG	CATCAGTGCCGTCAGTTCTTGT	204 bp
M-BCL6	CAGGAAGTTCATCAAGGCCAGT	TTTCTCAGTGGCATATTGTTCTCC	150 bp
M-Ghrl	AGAAAGGAATCCAAGAAGCCACC	GCCAACATCGAAGGGAGCATT	138 bp
M-Nlrp3	ATGACTTTCCAGGAGTTCTTCGC	CCAAAGAGGAATCGGACAACAA	185 bp
M-IL6	CATAGCTACCTGGAGTACATGAAGAA	GACTCCAGCTTATCTCTTGGTTGA	121 bp
M-GAPDH	CCTCGTCCCGTAGACAAAATG	TGAGGTCAATGAAGGGGTCGT	133 bp

Note：H：Human；M：mouse

Supplemental Table 3. A list of the CyTOF antibodies and reagents used in this research

Reagent or resource	Source	Identifier
Palladium ions	Fluidigm	N/A
Isothiocyanobenzyl-EDTA	Lumiprobe	M030
Fc receptor blocking reagent	BioLegend	422302
Helios mass cytometer	Fluidigm	Helios CyTOF
CD4	Fluidigm	3145002C
CD45	Fluidigm	3089005C
CD3e	Fluidigm	3152004C
CD86	Fluidigm	3172016C
F4/80	Fluidigm	3146008C
FoxP3	Fluidigm	3158003C
CD11b (Mac-1)	Fluidigm	3209003C
Ly-6G/C (Gr-1)	Fluidigm	3141005C
CD206 (MMR)	Fluidigm	3169021C
CD27	Fluidigm	3150017C
CD25 (IL-2R)	Fluidigm	3151007C
CD279 (PD-1)	Fluidigm	3159006C
CD274 (PD-L1)	Fluidigm	3153016C
CD161 (NK1.1)	Fluidigm	3170002C
CD152 (CTLA-4)	Fluidigm	3154008C
IFNg	Fluidigm	3165003C
CD223 (LAG-3)	Fluidigm	3174019C
CD14	Fluidigm	3156009C
CD21	Fluidigm	3168010C
TCRb	Fluidigm	3143010C
CD40	Fluidigm	3161020C
Granzyme B	Fluidigm	3171002C
FITC	Fluidigm	3160011C
APC	Fluidigm	3176007C
Cisplatin	Fluidigm	201064

Intercalator-Ir	Fluidigm	201192A
Maxpar cell staining buffer (500 ml)	Fluidigm	201068
Maxpar fix and perm buffer (100 ml)	Fluidigm	201067
Maxpar water (500 ml)	Fluidigm	201069
Maxpar cell acquisition solution plus for CyTOF XT-1L	Fluidigm	201244
Maxpar nuclear antigen staining buffer set	Fluidigm	201063

Supplemental Table 4. Rotator cuff tendon bone healing histological scoring system

Independent Parameter	Scoring Criteria (1–5 points)
Cellularity (inflammation)	1 = severe; 2 = moderate; 3 = mild; 4 = minimal; 5 = none
Proportion of fibroblastic cells (%)	1=<25；2=26-50；3=51-75；4=75-90；5=>90
Proportion of aligned cells (%)	1=<25；2=26-50；3=51-75；4=75-90；5=>90
Proportion of aligned fibers (%)	1=<25；2=26-50；3=51-75；4=75-90；5=>90
Matrix appearance	1 = very loose; 2 = loose; 3 = intermediate; 4 = dense; 5 = well-organized
TBI (tendon-bone interface) integrity	1=C(-),R(-),F(-),T(-)；2=C(+),R(-),F(-),T(-)；3=C(+),R(+),F(+),T(-)；4=C(+),R(+),F(+),T(+)
Continuity of the TBI (%)	1=<25；2=26-50；3=51-75；4=75-90；5=>90
Proportion of new bone formation (%)	1=<25；2=26-50；3=51-75；4=75-90；5=>90

Note: The scoring system includes 8 independent histological parameters, each graded from 1 to 5. The maximum possible score is 40, with normal native tissue serving as the reference control.

Abbreviations:

TBI = tendon-bone interface;

C = continuity;

R = regularity;

F = presence of fibrocartilage;

T = presence of tidemark.
